# Decoding Cell–Cell Interactions in Neurovascular Unit Co-Cultures Through Molecular Vibrational Spectroscopy and Biology

**DOI:** 10.3390/cells15201873

**Published:** 2026-10-15

**Authors:** Aleksandra Pragnąca, Agata Borkowska, Anna Antolak, Monika Leśniak, Karolina Augustyniak, Iga Czechowska, Kamilla Malek, Robert Zdanowski

**Affiliations:** 1 Faculty of Chemistry, Department of Chemical Physics, Jagiellonian University in Krakow, Gronostajowa 2, 30-387 Krakow, Poland; aleksandra.pragnaca@doctoral.uj.edu.pl (A.P.); anna.antolak@uj.edu.pl (A.A.); karolinakatarzyna.augustyniak@uj.edu.pl (K.A.); iga.czechowska@student.uj.edu.pl (I.C.); 2 Doctoral School of Exact and Natural Sciences, Jagiellonian University in Krakow, Prof. S. Lojasiewicza 11, 30-348 Krakow, Poland; 3 Laboratory of Molecular Oncology and Innovative Therapies, Military Institute of Medicine-National Research Institute, Szaserow 128, 04-141 Warsaw, Poland; aborkowska@wim.mil.pl (A.B.); mlesniak@wim.mil.pl (M.L.)

**Keywords:** neurovascular unit, 2D co-culture, blood–brain barrier, stress models, molecular biology, FTIR and Raman spectroscopy imaging, data mining

## Abstract

The blood–brain barrier (BBB) is a coordinated multicellular interface in which intercellular communication shapes responses to injury and metabolic stress. However, the contributions of individual cell types to collective stress adaptation remain poorly understood. Here, we examined molecular and metabolic responses of 2D neurovascular unit (NVU) co-culture models comprising endothelial cells, astrocytes, and pericytes exposed to hypoxia or sublethal HgCl_2_ toxicity. An integrated platform combining gene and protein expression profiling, label-free vibrational spectroscopic imaging, and machine-learning-based analysis was used to assess stress-induced changes in cell-cycle regulation and metabolism across mono- and co-culture configurations. Hypoxia broadly suppressed G_1_/S and G_2_/M regulators, inducing cell-cycle arrest with limited cell death, consistent with an adaptive response. HgCl_2_ caused heterogeneous, model-dependent changes in viability and proliferative capacity. Spectroscopic phenotyping revealed that the triple co-culture exhibited spectral features resembling those of pericyte monocultures, although the contributions of cell–cell interactions and differences in culture conditions could not be distinguished. Fluorescence imaging further revealed mitochondria-associated signals at cell–cell contact sites, suggesting a possible association of mitochondrial material with regions of close cellular interaction. The presence of pericytes was associated with a more stress-resistant cellular phenotype in the investigated co-culture configurations. The developed spectroscopic–molecular platform provides a label-free framework for characterizing cellular communication and stress responses in multicellular NVU co-culture models.

## 1. Introduction

The blood–brain barrier (BBB) is a highly specialized physiological interface that protects the central nervous system (CNS) from harmful substances, pathogens, and adverse systemic changes, while enabling the regulated exchange of essential nutrients and metabolites [1]. At its core, the BBB is formed by brain microvascular endothelial cells (BMECs), whose phenotype and function are dynamically regulated through interactions with pericytes, astrocytes, neurons, microglia, and the surrounding extracellular environment, collectively forming the neurovascular unit (NVU) [2]. Rather than functioning as an isolated endothelial barrier, the BBB is a multicellular system in which intercellular communication contributes to the regulation of junctional organization, transporter expression, metabolic support, and cellular responses to stress. Endothelial selectivity is supported by specialized junctional complexes involving claudins, occludin, and zonula occludens proteins, together with regulated influx and efflux transport systems such as GLUT1, P-gp, BCRP, and MRP1 [3,4]. Accordingly, understanding BBB-associated responses requires considering not only endothelial barrier properties but also the coordinated behavior of the NVU cellular components.

Dysfunction of the BBB and the broader neurovascular unit is associated with numerous neurological disorders, including stroke, epilepsy, Parkinson’s disease, and Alzheimer’s disease. These pathological states involve not only impaired endothelial barrier properties but also alterations in intercellular signaling, inflammatory regulation, metabolic support, and communication among endothelial cells, pericytes, astrocytes, and other NVU components. Disruption of this coordinated cellular network can contribute to altered permeability, neuroinflammation, and neuronal dysfunction, yet the mechanisms governing cell-specific and multicellular responses to injury remain incompletely understood [2]. Experimental investigation of these processes has traditionally relied heavily on in vivo animal models, which provide valuable insight into NVU complexity within a physiological environment. However, species-dependent differences in vascular phenotype and transporter expression, including differences in P-gp abundance between rodents and humans, can limit direct translation to human biology and influence drug pharmacokinetics and efficacy [3].

To overcome these limitations, in vitro BBB models have been developed, offering controlled, scalable, and ethically acceptable platforms for research. The most common approach involves Transwell-based systems employing semipermeable porous membranes, which are particularly suitable for assessing endothelial barrier properties, including paracellular permeability, transporter activity, and transendothelial electrical resistance (TEER). By separating luminal and abluminal compartments, these models enable quantitative assessment of solute transport across a confluent endothelial layer. However, conventional Transwell configurations do not reproduce physiological blood flow and shear stress and, depending on their design, may also restrict direct physical interactions between different NVU cell populations [5]. To better replicate in vivo conditions, dynamic microfluidic systems (BBB-on-chip) have also been introduced. These platforms incorporate three-dimensional (3D) architecture, simulated fluid flow, and co-cultures of various NVU cell types, enabling more physiologically relevant studies [6]. BBB-on-chip systems allow for real-time monitoring of barrier properties, such as ischemic response, inflammation, and neurotoxic exposure, often via integrated TEER sensors; however, they remain costly and technically demanding [5].

Another important advancement is the use of multicellular co-cultures in two- and three-dimensional (2D and 3D) formats. These models, including hydrogel-based systems, spheroids, and organoids, more accurately recreate the BBB’s spatial microenvironment and facilitate complex intercellular interactions. 2D co-cultures typically integrate primary astrocytes, pericytes, and endothelial cells to mimic the NVU, allowing detailed analysis of processes such as cytoskeletal remodeling and dynamic TEER modulation. In contrast, 3D models offer superior expression of tight junction proteins, enhanced responsiveness to external stimuli, and more physiologically relevant gene expression profiles [5]. The ability of 2D and 3D models to integrate with flow-based organ-on-a-chip systems further enhances their experimental potential, enabling comprehensive studies of BBB physiology and pathology [6]. Importantly, these models allow for both soluble factor-mediated signaling and direct cell–cell interactions, which are critical for barrier stability and function [2]. A particularly promising direction involves using Human-induced Pluripotent Stem Cells (hiPSCs), which can differentiate into multiple cellular components of the NVU. These systems offer a platform for personalized disease modeling and drug testing [7].

Collectively, these approaches highlight that no single in vitro BBB model can reproduce all structural, functional, and molecular features of the neurovascular unit. Instead, model configuration should be matched to the specific biological question being addressed [8]. Models optimized for quantitative assessment of barrier tightness, such as Transwell or microfluidic systems, are particularly valuable for TEER and permeability measurements, whereas other experimental questions require direct access to individual cells, subcellular structures, and heterotypic cell–cell interfaces [9]. This distinction becomes particularly important when investigating stress sensitivity, and metabolic responses, which can vary substantially between individual NVU cell types and may be further modified by their interactions within a multicellular system [10,11]. Moreover, compartmentalized configurations may restrict access to direct heterotypic cell–cell interfaces, while polymeric membrane or substrate materials can complicate spatially resolved in situ molecular imaging through substrate-derived spectral background and optical artifacts [12].

To address these questions, we employed a planar, direct-contact 2D co-culture system in which endothelial cells, astrocytes, and pericytes were maintained on the same surface. Rather than reproducing a continuous endothelial barrier separating luminal and abluminal compartments, the cultures were maintained under sub-confluent conditions to preserve spatially distinguishable cellular and subcellular regions. This configuration enabled direct heterotypic cell–cell interactions while providing optical access suitable for spatially resolved FTIR, Raman, and O-PTIR imaging. Accordingly, the model was not intended for TEER or transendothelial permeability measurements, which require formation of a continuous endothelial layer separating two compartments, but rather for detailed molecular phenotyping of individual NVU cell types and their interactions.

In the present study, we therefore investigated dual- and triple-cell-type 2D NVU co-cultures to characterize configuration-dependent differences in cell-cycle regulation, metabolic remodeling, and cellular responses to hypoxic and toxic stress. Targeted molecular analyses were integrated with label-free vibrational spectroscopic imaging and multivariate data analysis to characterize molecular changes at the level of individual cells, subcellular compartments, and intercellular contact regions. By combining conventional molecular assays with spatially resolved chemical imaging, this approach was designed to complement barrier-oriented NVU and BBB models and provide insight into cellular and molecular responses that may be obscured when barrier function alone is used as the principal experimental readout.

## 2. Materials and Methods

### 2.1. Cell Culture

Immortalized Human Brain Microvascular Endothelial Cells (HBMEC; Innoprot, Derio, Bizkaia, Spain), Human Astrocytes, and Human Brain Vascular Pericytes (HA and HBVP, respectively; ScienCell, Carlsbad, CA, USA) were cultured at 37 °C, 5% CO_2_. HBMECs were grown in Endothelial Cell Medium, HA in Astrocyte Medium, and HBVP in Pericyte Medium, each supplemented per the supplier’s protocols (ScienCell, Carlsbad, CA, USA). HBMECs were seeded on fibronectin-coated dishes (Thermo Fisher Scientific, Carlsbad, CA, USA), and HA and HBVP were seeded on poly-L-lysine surfaces (MilliporeSigma, Burlington, VT, USA). Cells were used at passage 5 and seeded at 10,000 cells/cm^2^. Co-cultures were established as HBMEC+HA (1:1), HBMEC+HBVP (1:1), and HBMEC+HA+HBVP (1:1:1), maintained in mixed media. For co-culture experiments, the respective cell types were mixed immediately before seeding and plated onto fibronectin-coated surfaces. Experimental cultures were intentionally maintained under sub-confluent conditions to enable spatially resolved analysis of individual cells and intercellular contacts. Co-cultures were allowed to establish for 24 h after seeding before stressor application and were then maintained for an additional 24 h under the respective control or stress conditions, resulting in a total co-culture period of 48 h. Hematopoietic contamination was excluded by the lack of CD45 expression.

For stress induction, cells were exposed to hypoxia (1% O_2_, BioSpherix, Parish, NY, USA) or 20 µM HgCl_2_ (MilliporeSigma, Burlington, VT, USA) for 24 h. HgCl_2_ was used as a model of sublethal heavy-metal neurotoxic stress. The 20 µM concentration was selected on the basis of preliminary dose–response experiments to induce measurable cellular stress while avoiding extensive cytotoxicity. Controls received matched conditioned medium; mercury exposure was performed in normoxic medium. Full details are provided in the Supplementary Materials.

### 2.2. RNA Isolation and RT-qPCR

RNA was extracted using a Direct-zol™ Miniprep kit (Zymo Research, Irvine, CA, USA) and quantified spectrophotometrically. cDNA was synthesized with a High-Capacity cDNA Reverse Transcription kit (Invitrogen, Carlsbad, CA, USA) from 1–1.5 µg RNA. qPCR was performed with SYBR Green PCR Master Mix (Thermo Fisher Scientific, Carlsbad, CA, USA) on a CFX Connect Real-Time System (Bio-Rad Laboratories, Hercules, CA, USA). Genes analyzed included TP53, RB1, RBL1, CDKN1B, CCND1, CCNE2, CCNA2, CDK1, CCNB1, TJP1 (ZO-1), TJP3, CLDN1 normalized to GAPDH. Primer sequences are presented in Table S1. Relative expression was calculated by the 2^−ΔΔCt^ method, then log-transformed and presented relative to the control. Full details are provided in the Supplementary Materials.

### 2.3. Western Blotting

Proteins were extracted in RIPA buffer containing inhibitors and quantified using a BCA Protein Assay kit (Thermo Fisher Scientific, Carlsbad, CA, USA), separated on 4–20% SDS-PAGE gels, and transferred to PVDF membranes (Bio-Rad Laboratories, Hercules, CA, USA). After blocking with non-fat milk, membranes were incubated with antibodies against Cyclin D1, pRb, p27, p21, Cyclin A2, Cyclin B1, p-CDK1 Tyr15, GFAP, and GAPDH, cf. Table S2. HRP-conjugated secondary antibodies (Vector Laboratories, Newark, CA, USA) and chemiluminescence were used for detection. Bands were quantified with ImageJ software (version 1.53). Full details are provided in the Supplementary Materials.

### 2.4. Flow Cytometry

Cells harvested with Accutase (Thermo Fisher Scientific, Carlsbad, CA, USA) were stained with fluorophore-conjugated antibodies (CD31, CD140b, CD105, CD45, P-gp,), cf. Table S3. Next, cells were fixed in 4% formaldehyde and analyzed on a CytoFLEX flow cytometer (Beckman Coulter, Brea, CA, USA). Cellular debris and CD45^+^ events were excluded. At least 10,000 events/sample were acquired. Full details are provided in the Supplementary Materials.

### 2.5. Apoptosis Assay

Apoptosis was measured using a FITC Annexin V/PI staining kit (BD Pharmingen, San Jose, CA, USA). Attached and detached cells were pooled, washed, stained, and analyzed by flow cytometry. Early apoptotic, late apoptotic, and necrotic cells were quantified. Full details are provided in the Supplementary Materials.

### 2.6. Cell Cycle Analysis

Cell cycle distribution was determined using a Tali™ Cell Cycle kit (Thermo Fisher Scientific, Carlsbad, CA, USA). Cells were fixed in 70% ethanol, stained with the manufacturer’s reagent, and analyzed by flow cytometry. DNA histograms were processed with FlowJo software (version 10.9) (BD Life Sciences, Ashland, OR, USA) to determine G_1_, S, and G_2_ fractions. Full details are provided in the Supplementary Materials.

### 2.7. Live Fluorescence Staining of Mitochondria

HBMECs, HAs, and HBVPs, upon reaching 80–90% confluence, were stained with 150 or 200 nM MitoTracker (Orange CMTMRos—HBMEC; Green FM—HA; Red FM—HBVP; Thermo Fisher Scientific, Carlsbad, CA, USA). The MitoTracker dyes were diluted in the culture medium appropriate for each cell type. Cells were incubated at 37 °C in a 5% CO_2_ atmosphere for 1 h. Following staining, cells were harvested and seeded in the triple coculture on Chamber Slides (Thermo Fisher Scientific, Carlsbad, CA, USA). Immediately after seeding, half of the culture vessels were transferred to a hypoxia chamber, while the remaining ones were maintained under normoxic conditions. After 24 h, nuclei were stained with bisbenzimide H33342 trichloride (Sigma-Aldrich, Burlington, VT, USA) for 15 min. at 37 °C. Subsequently, cells were fixed with 4% PFA for 15 min at 37 °C. For microscopic observation, slides were sealed with Immunofluorescence Mounting Medium (Dako, Glostrup, Denmark). Images were acquired using a Zeiss Axio Observer 7 fluorescence microscope at 63× magnification and analyzed with Zen Blue v. 3.4 software (Zeiss, Oberkochen, Germany). Negative controls were processed in parallel. Full details are provided in the Supplementary Materials.

### 2.8. Raman, FTIR, and O-PTIR Spectroscopy Imaging

Spectroscopic data collection and analysis were performed across three independent biological experiments. In each experimental group, mono- or multi-cultures, two technical replicates were prepared by seeding cells on two CaF_2_ windows.

Raman imaging was conducted using a WITec Alpha 300 confocal Raman microscope (WITec, Ulm, Germany) under 532 nm and a 40× water-immersion objective (W Plan-Apochromat VISIR, NA 1.0, Zeiss, Oberkochen, Germany) with an 1800 grooves/mm diffraction grating. The nominal laser power was set to 40 mW. Because the measurements were performed using a water-immersion objective, the laser power was specified at the laser source rather than directly at the sample plane. The Raman spectral axis was calibrated using a silicon standard, based on its characteristic Raman band at approximately 520 cm^−1^. Spectra were acquired with an integration time of 0.5 s and a spectral resolution of 3 cm^−1^. Maps were collected with an x-y grid with a step size of 1 μm. 20 cells were measured per technical replicate. Finally, 36 single-cell Raman maps were manually selected from different biological replicates for further analysis based on appropriate cell morphology, absence of visible artifacts, and high signal-to-noise ratio.

After Raman data acquisition, the cells were washed three times with distilled water, air-dried, and subsequently subjected to FTIR imaging. An Agilent 670-IR spectrometer, combined with a 620-IR microscope, was used for IR spectroscopy imaging (Agilent, Santa Barbara, CA, USA). The focal plane array (FPA) detector consisted of a 16,384-pixel matrix arranged in a 128 × 128 grid (field of view: 700 μm × 700 μm). From each CaF_2_ window, 3–4 FTIR images were captured, with around 10–15 cells per image. Averaged FTIR spectra for individual cells were extracted using a Python script (version 3.10.0), excluding spectra with distortions or low signal-to-noise ratios. To maintain balanced group sizes, 100 spectra were randomly selected for each experimental group. In this setup, biological and technical replicates, fields of view, cells, and spectra formed nested observational units within each biological replicate and were not treated as independent replicates. FT-IR spectra and background were recorded using 128 co-added scans. Spectral data were collected in the 3700–900 cm^−1^ range with a spectral resolution of 4 cm^−1^. Optical Photothermal Infrared spectroscopy (O-PTIR) measurements were conducted using a mIRage-LS O-PTIR microscope (Photothermal Spectroscopy Corporation, Santa Barbara, CA, USA) operated in the co-propagation mode. The instrument uses pulsed quantum cascade lasers (QCLs; MIRcat 2400, Daylight Solutions (San Diego, CA, USA)) as the IR pump source and a continuous-wave 532 nm laser as the optical probe beam. The instrument is equipped with 40× reflective Cassegrain objective. O-PTIR spectra were acquired using the available QCL spectral ranges of 1000–1850, 2000–2400, and 2700–3000 cm^−1^, with a spectral resolution of 2 cm^−1^. We conducted O-PTIR measurements after hyperspectral FTIR imaging on air-dried cells from two technical replicates. Single-wavenumber O-PTIR chemical images of an approximately 200 µm × 100 µm area were acquired at 1240, 1740, and 2850 cm^−1^ with a 1 µm step size as in Raman imaging. We selected ROIs to measure 4–7 cells simultaneously. The O-PTIR ratio images for 1240/1740 cm^−1^ and 1740/2850 cm^−1^ were used to spatially identify cellular regions of interest (ROIs) corresponding to the nucleus and lipid-rich regions, respectively. The ratio maps provided spatial contrast to distinguish these cellular compartments, and we identified ROIs manually based on the distribution of the corresponding ratio signal and cellular morphology. Based on these ratios and their highest intensities, we collected hyperspectra from the nucleus and lipid-rich regions in three cells.

Spectral data sets were analyzed using a combination of software packages, including WITec Project (version 5.0, WITec, Ulm, Germany), CytoSpec (version 2.00.01, CytoSpec, Berlin, Germany), MatLab (R2020a, MathWorks, Natick, MA, USA), PLS_Toolbox (version 9.2.1, Eigenvector Research, Manson, WA, USA), OPUS (version 7.2, Bruker, Billercia, MA, USA), and Origin 9.1 (version 2020b, OriginLab Corporation, Northampton, MA, USA).

Briefly, after preprocessing, Raman maps of the single cells were subjected to Multivariate Curve Resolution (MCR) and k-means Cluster Analysis (KMCA) to resolve intracellular structures and compare their distribution and alternation across in vitro models and stress conditions. FTIR spectra of the entire cell body were analyzed by using Principal Component Analysis (PCA) and cell phenotyping modeled with the help of Partial Least Squares Discriminant Analysis (PLS-DA) and Support Vector Machine (SVM). High-resolution O-PTIR maps were analyzed to extract IR information from physically interacting cells to complement the corresponding Raman maps. All details regarding data preparation for these analyses are provided in the Supplementary Materials.

## 3. Results

### 3.1. Cell Models and Their Phenotyping

**Figure 1:**
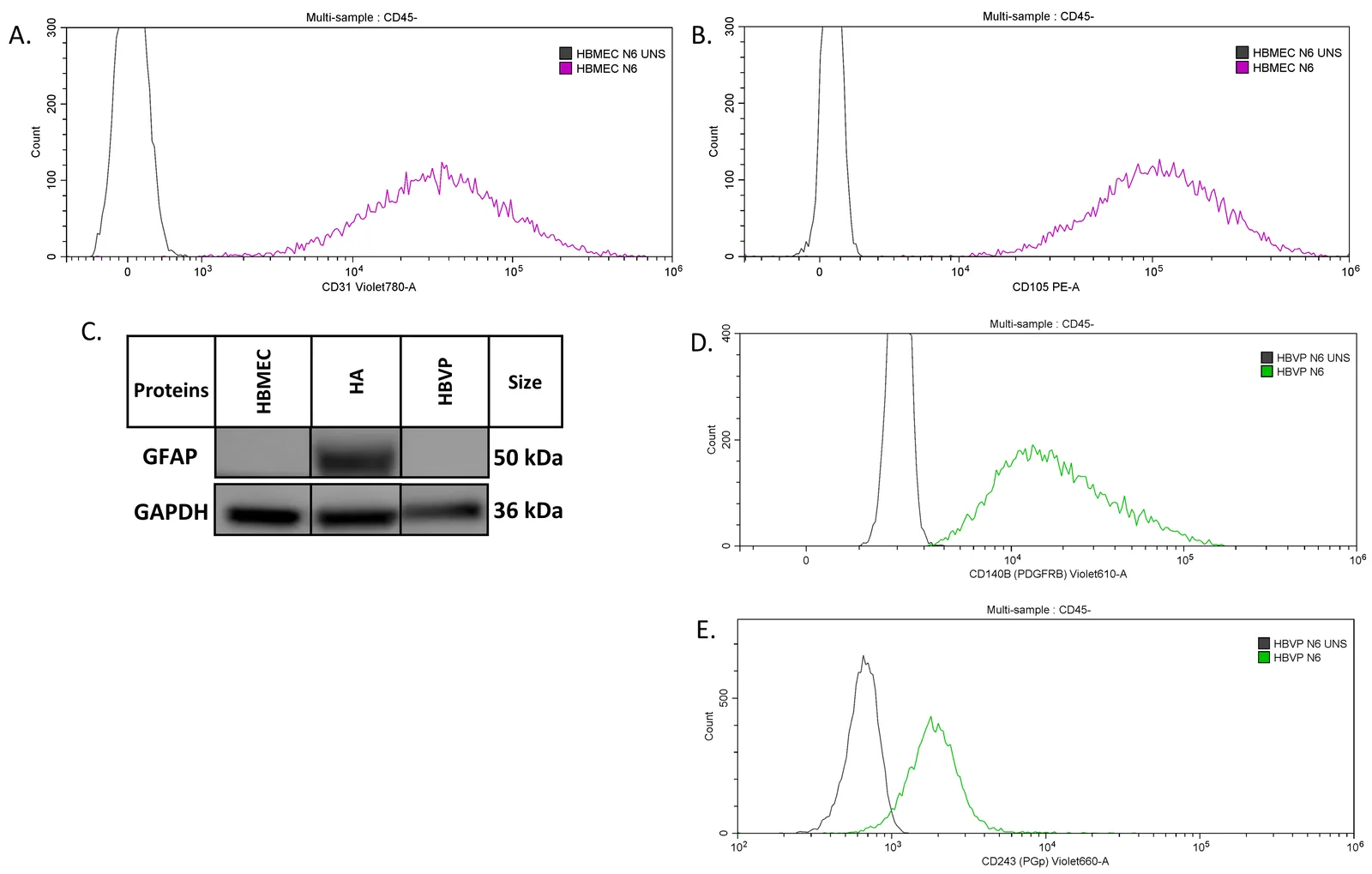
Molecular characterization of NVU cell lines based on the expression of cellular markers for: HBMEC (**A**,**B**) surface expression of CD31 and CD105 (magenta) vs. unstained control (grey) measured with flow cytometry; HA (**C**) GFAP expression measured with Western Blot, where GAPDH serves as loading control; HBVP (**D**,**E**) HBVP—CD140b and CD243 (green) vs. unstained control (grey) measured with flow cytometry. Data shown are representative of n = 3 experiments.

To investigate cellular interactions within a simplified neurovascular unit environment, we employed planar 2D culture systems comprising HBMEC, HA, and HBVP. Endothelial cells were included in all co-culture configurations, whereas astrocytes and pericytes were added to generate double (HBMEC+HA and HBMEC+HBVP) and triple (HBMEC+HA+HBVP) cellular systems. The triple co-culture incorporated the three principal cellular components examined in this study and was used to assess how increasing cellular complexity influences phenotype, metabolic state, and stress responses. Importantly, these cultures were designed to investigate direct cellular interactions and were not intended to reproduce or quantitatively assess endothelial barrier tightness. The phenotypic identity of each cell type was established by combining flow cytometry and Western blot analysis, using well-established surface and intracellular markers. Importantly, this panel was deliberately restricted to a single canonical surface marker per cell type—a choice that mirrors routine validation practices in the BBB field and as discussed below, highlights the inherent limitations of single-marker immunophenotyping in complex multicellular models. HBMEC expressed both the endothelial adhesion molecule PECAM-1 (CD31) and TGF-β accessory receptor endoglin (CD105) [13] (Figure 1A,B). HA identity was supported by Western blotting for glial fibrillary acidic protein (GFAP), a canonical intracellular intermediate filament protein expressed in astrocytes [14,15] (Figure 1C). The majority of HBVPs were positive for platelet-derived growth factor receptor-β (PDGFR-β, CD140b) (Figure 1D). PDGFR-β is a widely used pericyte marker in the CNS microvasculature [16,17], and its strong expression here aligns with prior descriptions of brain pericytes. A subset of the HBVP surface also expressed the drug efflux transporter P-gp (P-glycoprotein ABCB1) (Figure 1E).

CD31 and CD140b were used for flow-cytometric phenotyping of HBMEC and HBVP in the co-culture systems (Figure S1A–C). Astrocytes were therefore operationally defined as CD31^−^ cells in HBMEC+HA co-cultures and as CD31^−^CD140b^−^ cells in HBMEC+HA+HBVP co-cultures, while their presence was independently supported by Western blot analysis of GFAP expression (Figure S1G). These immunostaining results revealed that CD31^+^ HBMEC comprised ca. 60% and 45% in the double and triple co-cultures, respectively (Figure S1D–F). HA constituted ~40% of HBMEC+HA and ~25% of HBMEC+HA+HBVP, whereas HBVP was detected at comparable levels (~20–25%) in all cellular systems (Figure S1D–F).

**Figure 2:**
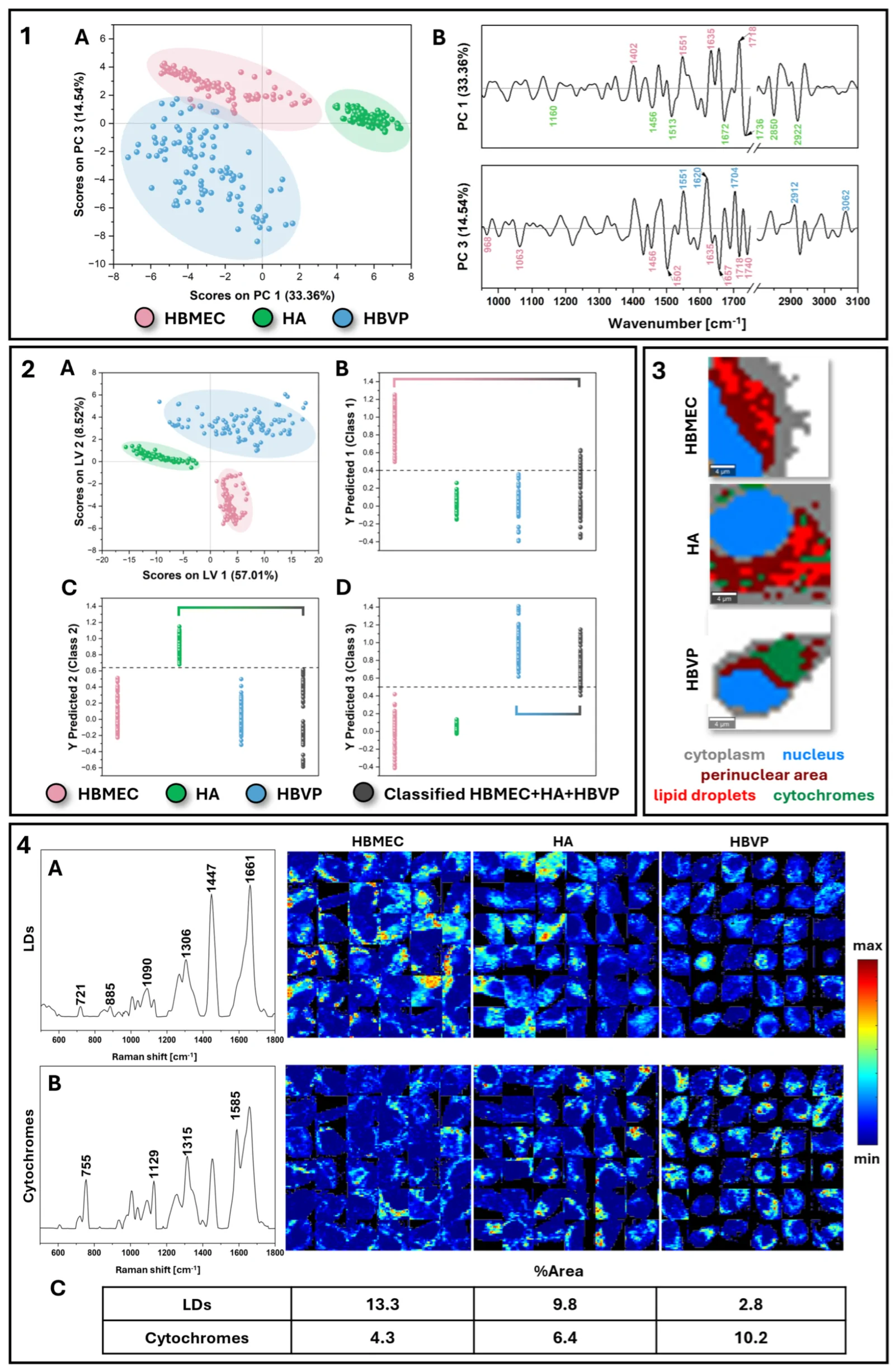
(**1**,**2**) Cell phenotyping based on FTIR spectra (n = 100 per group) of the single cells calculated using PCA with (**1A**) scores and (**1B**) loadings plots; and PLS-DA with (**2A**) LV scores plot from classification of the cells from the monocultures (n = 100 per group) and one-against-all classification of HBMEC+HA+HBVP (n = 200) to (**2B**) HBMEC, (**2C**) HA, and (**2D**) HBVP. (**3**) Single-cell compartmentation based on KMCA of the Raman images. (**4**) MCR components (left) and their distribution (right) calculated from Raman images (n = 36 per group) of the monocultures corresponding to (**4A**) lipid droplets and (**4B**) cytochromes with (**4C**) their % contribution to the cellular area.

To further characterize the junction-associated molecular phenotype of the investigated cultures, we assessed the expression of TJP1, TJP3, and CLDN1. Representative transmitted-light images confirmed the intentionally sub-confluent organization of all mono- and co-culture configurations, consistent with the spectroscopic workflow (Figure S2A). Under control conditions, TJP1 and CLDN1 transcripts were detected across all cellular models investigated, whereas TJP3 showed lower, more restricted expression and was not quantifiable in HA or HBVP monocultures (Figure S2B). These findings indicate junction-associated molecular markers within the experimental system, but do not imply formation of a continuous barrier-forming endothelial monolayer. This single-marker approach exposes the ambiguity inherent in conventional immunophenotyping—where no single surface marker unequivocally and exclusively identifies each NVU cell type—so we complemented it with a machine learning (ML) method that leverages single-cell FTIR imaging as a marker-independent alternative. Chemical images provide the molecular fingerprint of the biological specimen, which is encoded in their spectra and sensitive to the cell type and its metabolism [18,19]. Principal Component Analysis (PCA), an unsupervised data-mining method for exploring data and visualizing its distribution, was first applied to the FTIR spectra of the cell body of the monoculture NVU cells (Figure 2(1) and Figure S3). The scores plot showed a clear-cut discrimination between BBB cell lines along PC-1 and PC-3, with a total variance of 48%. PC-1 segregated the highly uniform spectral HA from the others because of discriminators attributed to esterified lipids (1160 and 1736 cm^−1^), long fatty acid acyl chains (2850 and 2922 cm^−1^), and the Tyr amino acid residue (1513 cm^−1^) (Figure 2(1B)). HBMEC and HBVP clustered along PC-3, and the single cells were more dispersed across their respective clouds, indicating their biochemical diversity (Figure 2(1A)). HBMEC showed main score vectors at 1657 and 1635 cm^−1^ (α-helical and β-sheet secondary protein structures, respectively) and at 968 and 1240 cm^−1^ (DNA), along with signals for phospholipids (1063 cm^−1^) and non-esterified fatty acids (1402 and 1718 cm^−1^). In turn, HBVP-specific protein markers appeared at 1551, 1620, 2912, and 3062 cm^−1^ (Figure 2(1B)).

Subsequently, an ML approach incorporating multiclass PLS-DA (Partial Least Squares Discriminant Analysis) and SVM (Support Vector Machines) was applied to determine whether these unique spectral profiles enable classification of NVU cells. Model 1 was built using monocultured cells to confirm PCA grouping. The classification performance, including accuracy, specificity, and sensitivity (0.95–1.0), as well as confusion matrices, was comparable between the methods, as seen in Tables S4 and S5 and Figure S4. In addition, the latent variable (LV) plot of PLS-DA exhibited a pattern like the PCA scores plot (Figure 2(1A,2A)). The overall agreement between the SVM and PLS-DA results independently confirmed the observed cell-type discrimination and supported the consistency of the observed spectral-level classification pattern across two classification algorithms on FTIR spectra of the single cell body.

Model 2 used monoculture cells for calibration (n = 100 cells/group), while FTIR spectra from the HBMEC+HA+HBVP culture were used as an unclassified prediction set to assess differences in spectral phenotypes associated with the co-culture configuration. We applied a “one-against-all” PLS-DA model to two datasets. First, we classified another set of FTIR spectra from monocultures of HBMEC, HA, and HBVP (n = 100 cells/group) to validate the model; we assigned each cell type to the appropriate group, confirming that the FTIR profile is specific to each group (Table S4). Next, we used 200 HBMEC+HA+HBVP spectra from all biological replicates for external prediction. As a result, 21% of the co-cultured cells were identified as HBMEC (Figure 2(2B)), whereas none were assigned to HA (Figure 2(2C)). Notably, the HBVP-against-all classification identified 98% of co-cultured cells as HBVP (Figure 2(2D)). We also tested the same set of HBMEC+HA+HBVP cells using SVM (Figure S4). The prediction probabilities showed a similar assignment trend to PLS-DA, strengthening this observation. Overall, these results indicate a pronounced convergence of the spectral features of the HBMEC+HA+HBVP cells toward the HBVP profile. We interpret this finding as a shift in the overall spectral profile of the triple co-culture toward the spectral domain associated with the HBVP reference class, rather than evidence of transdifferentiation or loss of the underlying endothelial and astrocytic identities. Such spectral convergence may reflect changes in the cellular microenvironment and community-level interactions under co-culture conditions, including media effects and other model-specific factors.

Sub-micron lateral resolution in Raman images is widely used to visualize cellular compartments and their biochemical composition by clustering algorithms that adjust individual hyperparameters to cellular structures, as shown in the false-color KMCA maps in Figure 2(3) [20]. Rather than a conventional data-reduction approach, we employed MCR to identify unique NVU cell features in Raman images of multiple cells simultaneously. This method decomposed the image pixel spectra into constituent components, yielding a set of selective images for each constituent, along with its corresponding pure spectral profile. Seven MCR components decomposed the Raman images into typical cellular compartments (Figure S5). Components corresponding to Raman profiles of lipid droplets (LDs) and mitochondrial cytochromes, with multiple specific bands, showed the most pronounced differences among analyzed cell lines (Figure 2(4A,B)) [21]. Previous studies have validated the cytochrome Raman profile as a mitochondrial marker and its correlation with cellular metabolic activity, but we need to emphasize that this detection should not be treated as a direct functional assay of mitochondrial activity [22]. The quantitative image analysis revealed a marked accumulation of LDs, enriched in phospholipids and unsaturated fatty acids, within HBMECs (13% of the cellular area), compared with HBVP (3%), cf. Figure 2(4C). HA also contained LDs in nearly all cells, although their abundance was lower than in HBMECs (ca. 10%). Conversely, the cytochrome signal reached its highest intensity in HBVP, covering 10% of the cellular area, indicating distinct distributions of cytochrome-associated Raman signals across the investigated cell types (Figure 2(4B,C)).

### 3.2. The Effect of Hypoxia and Neurotoxicity on the NVU Co-Culture Models

The mono- and co-cultured NVU systems were exposed to hypoxic conditions (1% O_2_) or to induce sublethal heavy-metal neurotoxic stress and were treated with 20 µM HgCl_2_ for 24 h. Importantly, these treatments were designed to trigger metabolic and regulatory alterations without causing overt cell loss. The relative proportions of each cell type within the co-cultures remained stable compared to normoxia, and no cell type was selectively lost or overrepresented during the 24 h exposure (Figure S1D–F). This indicated that the observed changes reflect stress-induced alterations in cell physiology rather than disproportionate cytotoxicity or loss of a specific population.

### 3.3. Cellular Response to Stress

**Figure 3:**
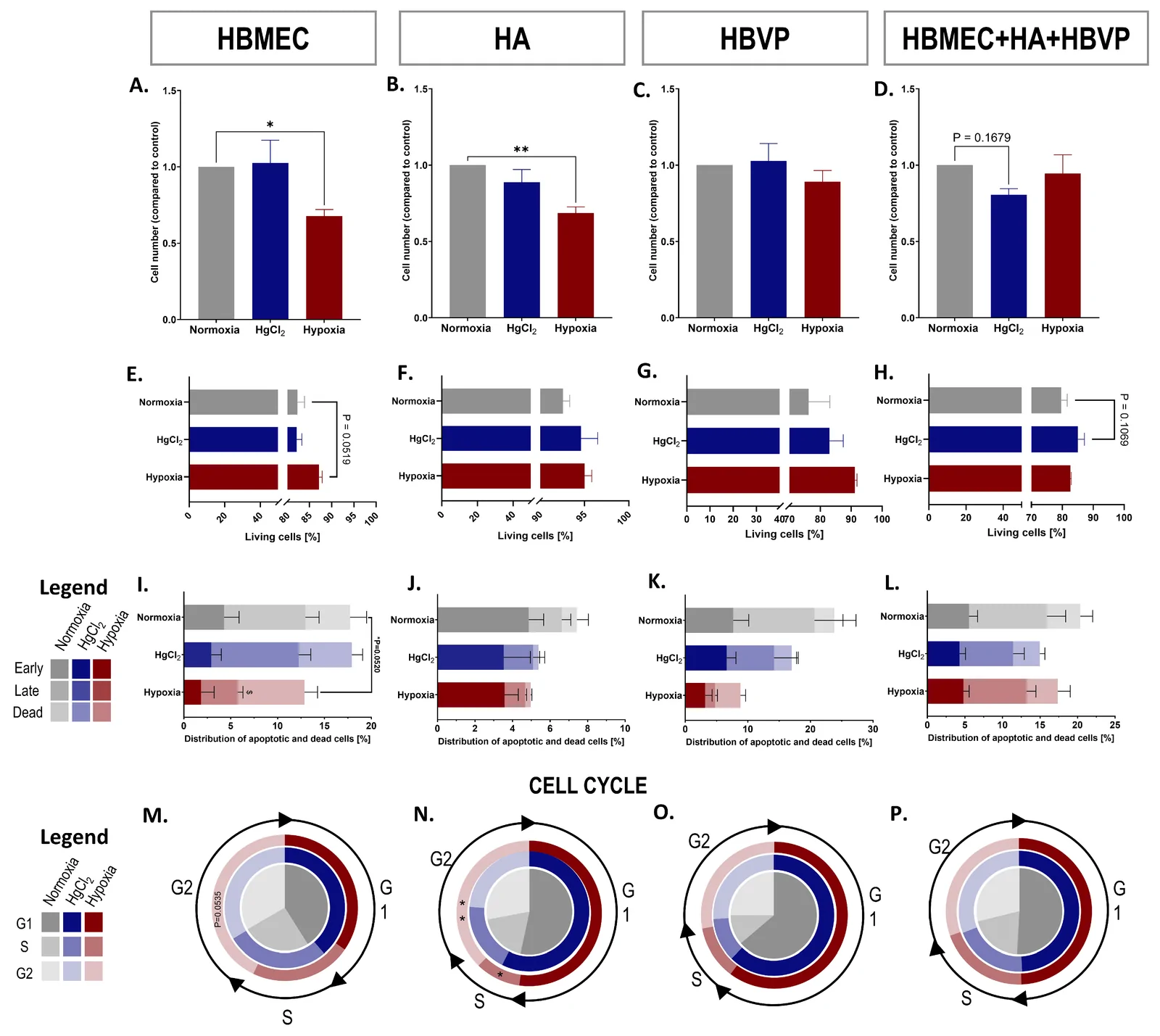
Effects of 24 h exposure to 20 µM HgCl_2_ and 1% O_2_ (hypoxia) on cell number; n = 4 (**A**–**D**), viability (**E**–**H**), n = 3, apoptosis ((**I**–**L**); populations of early and late apoptotic, and dead cells) n = 3, and cell-cycle ((**M**–**P**); DNA content in G_1_, S, and G_2_ phases; n = 4) in the BBB monocultures and triple co-culture. Statistical significance to the normoxic control: * *p* < 0.05, ** *p* < 0.01. Statistical significance to normoxic control for late apoptosis: $ *p* < 0.05. Each result is presented as mean ± SEM, except for cell cycle distribution, where results are presented as mean.

The first effect of the stress was observed in the total cell count. Under hypoxia, the cell counts of HBMEC and HA, and HBMEC+HA significantly decreased, in contrast to HBVP, HBMEC+HBVP, and HBMEC+HA+HBVP (Figure 3A–D and Figure S6A). HgCl_2_ reduced total cell number only in HBMEC+HA+HBVP (Figure 3D). No significant differences in cell viability and the accumulation of apoptotic/dead cells were detected in HBVP, HBMEC+HBVP, and HBMEC+HA+HBVP upon hypoxia (Figure 3C,G,H and Figure S6F,G). HgCl_2_ exposure did not significantly alter viability across cultures, except to increase it in HBMEC+HA+HBVP (Figure 3H). Interestingly, despite the reduction in the cell count, the hypoxia-exposed HBMEC, HA, and their co-culture showed higher viability and lower apoptosis/death rate than in normoxia (Figure 3E,F,I–L and Figure S6B,C). This paradoxical increase in viability suggested that oxygen depletion did not cause massive cell death but rather slowed proliferation or arrested cell-cycle progression, resulting in fewer new cells, while most cells remained viable.

The cell-cycle distribution results confirmed that hypoxia primarily induced growth arrest rather than cell death, as evidenced by alterations in the populations of the G_1_, S, and G_2_ phases. Specifically, accumulation of cells in the G_2_ phase was observed in HBMEC and HA (Figure 3M,N), indicating a cell-cycle arrest prior to mitosis, like that observed in HBMEC+HA, although this was not statistically significant (Figure S6D). Astrocytes also exhibited a reduced proportion of cells in S phase, further supporting an overall slowdown in cell-cycle progression (Figure 3N). In contrast, HBVP and HBMEC+HBVP once again were resistant to both stress agents (Figure 3O,P and Figure S6H). Altogether, these analyses indicated that hypoxic stress had a more pronounced effect on NVU cell proliferation than HgCl_2_. A relatively short-term exposure to 1% O_2_ led to a marked cell-cycle arrest, particularly in endothelial cells and astrocytes, whereas HgCl_2_ produced only a modest effect, detectable only in the triple co-culture setting. HBVP exhibited relatively limited changes in cell number and cell-cycle distribution under the investigated stress conditions. Moreover, pericyte-containing co-cultures, particularly HBMEC+HBVP, showed greater preservation of cell number and cell-cycle profiles under hypoxia than HBMEC+HA, suggesting an association between the presence of pericytes and a more stress-resistant co-culture phenotype. Because viability and cell-cycle outcomes in the mixed cultures were assessed at the culture level rather than separately for each constituent cell type, these data do not identify which specific population accounts for the observed differences.

**Figure 4:**
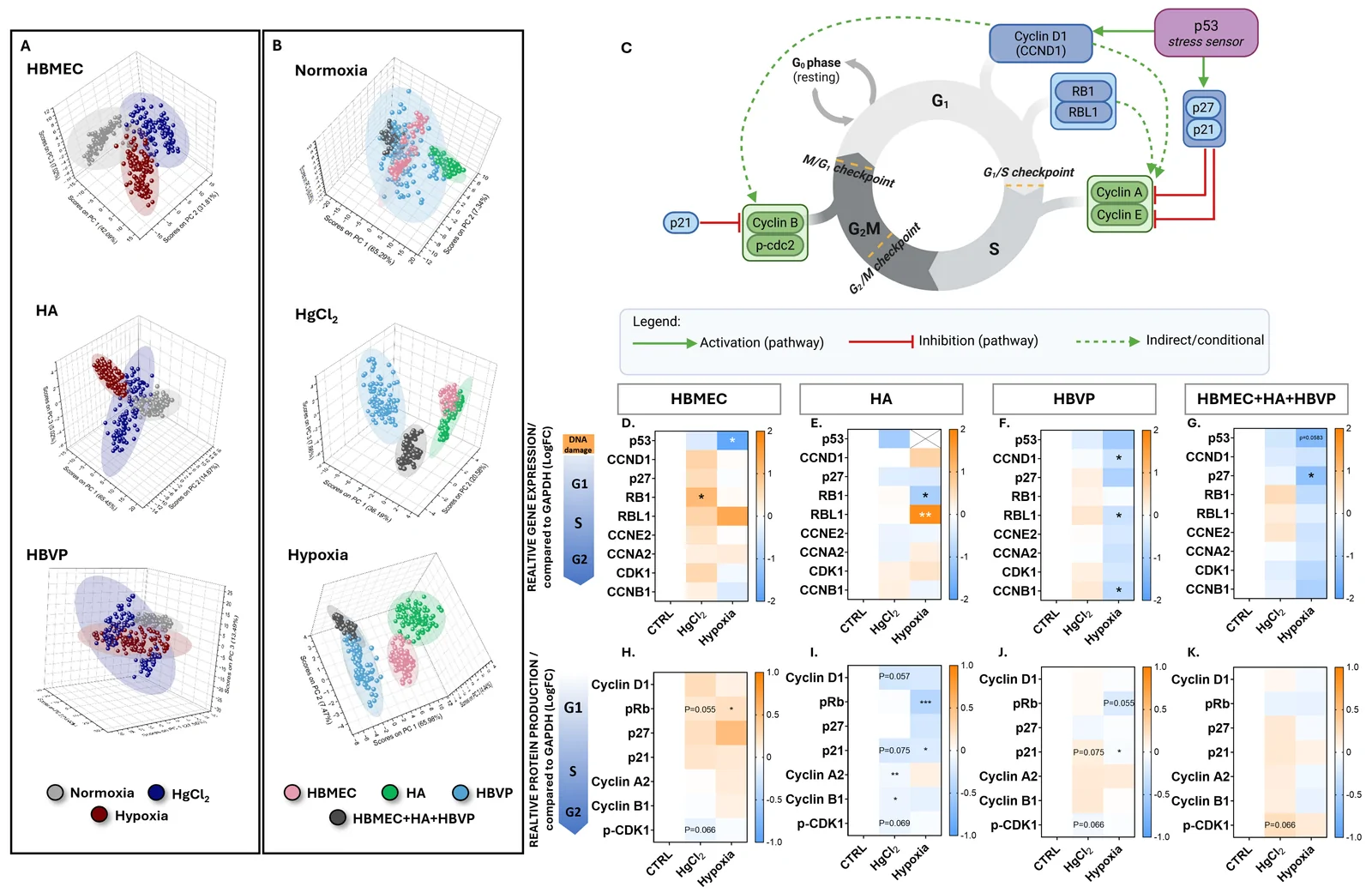
IR phenotyping based on the FTIR PCA of the NVU cells in normoxia and upon stress due to exposure to HgCl_2_ and hypoxia in (**A**) monocultures and (**B**) monocultures with triple co-culture. The score plots were generated from whole-cell FTIR spectra of 100 cells per group. Each point corresponds to one cell. (**C**) The schematic illustrates the main phases of the cell cycle (G_0_, G_1_, S, G_2_/M) and the key checkpoints (G_1_/S and G_2_/M), highlighting the molecular regulators investigated in this study. Cyclins (Cyclin D1/CCND1, Cyclin E, Cyclin A, Cyclin B and p-cdc2) together with retinoblastoma family proteins (RB1, RBL1) govern cell cycle progression by controlling phase transitions. The tumor suppressor p53 functions as a cellular stress sensor and induces the expression of cyclin-dependent kinase inhibitors (p21 and p27), which suppress cyclin–CDK complexes and lead to cell cycle arrest (this schematic was created with BioRender.com). Heatmaps of (**D**–**G**) mRNA fold-changes presented as logFC of p53, CCND1, p27, RB1, RBL1, CCNE2, CCNA2, CDK1, and CCNB1, n = 4 and (**H**–**K**) protein fold-changes in cyclins D1, A2, and B1, pRb, p27, p21, and p-cdc2 in the monocultures and triple co-culture n = 3. Statistical significance for the normoxic control: * *p* < 0.05, ** *p* < 0.01, *** *p* < 0.001. Each box represents the mean value from n ≥ 3 experiments.

Metabolic reprogramming was evaluated using IR/PCA phenotyping of the whole cellular body (Figure 4 and Figure S7). HBMEC and HA formed distinct groups, indicating higher sensitivity to stressors, unlike HBVP (Figure 4A). In the latter, stressed cells were clustered closely together, reflecting their relative resistance. Next, PCA revealed distinct behavior in the co-cultured cells compared with the monocultures. Under normoxic conditions, the HBMEC+HA co-culture exhibited a distinct spectral profile compared with the corresponding monocultures (Figure S7). Furthermore, HBVP-containing co-cultures displayed spectral features resembling those of the HBVP monoculture, a pattern that was also observed under hypoxic conditions (Figure 4B and Figure S7). For HgCl_2_, HBMEC+HA overlapped HBMEC, whereas HBMEC+HBVP and HBMEC+HA+HBVP formed separate clusters (Figure 4B and Figure S7).

### 3.4. Stress Response at the Gene and Protein Level

To characterize the molecular mechanisms associated with stress-induced changes in cell-cycle progression, we examined a panel of key cell-cycle regulators at the gene and protein levels. These include proteins that act as brakes on cell division (p53, p21, p27), proteins that release those brakes and allow progression (Cyclin D1, pRb phosphorylation), proteins that drive DNA replication (Cyclin E2, Cyclin A2), and proteins that trigger mitosis (Cyclin B1, CDK1). Together, these regulators span the full cell cycle—from the decision to divide (G_1_ phase) through DNA copying (S phase) to cell splitting (G_2_/M phase) (Figure 4C).

The most striking finding was that the triple co-culture—the most complex co-culture system examined in this study—showed the most restrained and coordinated molecular response to both hypoxia and HgCl_2_ (Figure 4G). Only two regulators, TP53 and CDKN1B, changed, and both decreased. This pattern is consistent with a controlled adaptive pause rather than a stress-driven breakdown: the cell cycle slows, but the molecular machinery remains intact and poised for recovery.

Simpler models—monocultures and double co-cultures—showed broader dysregulation. Hypoxia reduced TP53 transcript levels in HBMEC, HA, and HBMEC+HA cultures, while the G_1_ brake proteins p21 and p27 decreased in HA and HBVP under hypoxia (Figure 4D,E and Figure S8). The gate-keeping protein pRb, which normally prevents premature entry into DNA replication, increased in stressed HBMEC but fell in hypoxic HA, HBVP, and HBMEC+HBVP (Figure 4H–J and Figure S8), suggesting that simpler systems lose coherent checkpoint control under stress.

DNA replication regulators Cyclin E2 and Cyclin A2 were specifically suppressed under hypoxia in HBMEC+HBVP (Figure S8C), pointing to S-phase arrest in the double pericyte co-culture that was absent from the triple model. G2/M-associated regulators were also affected by stress. Cyclin B1 declined in hypoxic HBVP and in HgCl_2_-treated HA, whereas inhibitory phosphorylation of CDK1 at Tyr15 decreased across several mono- and double-culture conditions. In contrast, Tyr15-phosphorylated CDK1 increased in the triple co-culture following HgCl_2_ exposure (Figure 4H–K), indicating distinct regulation of the G_2_/M checkpoint in this culture configuration.

To determine whether the molecular response to stress also involved junction-associated features, we additionally assessed the expression of TJP1, TJP3, and CLDN1 (Figure S2C). TJP1 expression remained comparatively stable across most culture configurations following HgCl_2_ exposure or hypoxia. In contrast, TJP3 and CLDN1 showed more variable, model-dependent responses, including decreases in selected culture configurations. However, none of these stress-induced changes remained statistically significant after correction for multiple comparisons. TJP3 was not quantifiable in HA or HBVP monocultures and showed limited quantifiable replicate numbers in some co-culture conditions.

Taken together, these data show that increasing co-culture complexity was associated with distinct patterns of molecular stress responses, particularly in cell-cycle regulation. The triple co-culture exhibited a more restricted pattern of cell-cycle-related changes than several mono- and double-culture configurations, whereas the expression of the investigated junction-associated genes was comparatively stable or showed model-dependent variability without significant stress-induced changes.

### 3.5. Raman Markers of Non-Contact Cell–Cell Communication and Stress Response

To gain insight into cellular responses to their microenvironment via intercellular communication and stress adaptation, we again used Raman-based MCR analysis, selecting cells that were not physically in contact with one another. Raman profiles of spatially separated cells were examined to characterize molecular heterogeneity within the co-culture environment. Such differences may be associated with soluble factor-mediated signaling, although the contribution of other culture-related factors cannot be excluded.

Under normoxic conditions, MCR analysis resolved distinct component classes that could be assigned to specific cellular regions, including the nucleus (characterized by nucleic acid–associated Raman bands), the cytoplasm (rich in protein signals), the perinuclear region enriched in proteins and phospholipids (endoplasmic reticulum), small intracellular LDs, and a cytochrome-rich class corresponding to mitochondria (Figure S9). In addition, MCR segregated granular structures in the nucleus and was characterized by Raman signals attributed to proteins and cytosine and guanine bases (nucleoprotein class in Figure S9). The distribution of normalized values of each MCR component in the image pixels indicated that HBMEC+HA+HBVP possessed LDs, which were abundant in HBMEC and HA, and mitochondria, which were specific to HBVP (Figure 2(4)). HBVP features in the cytoplasm, perinuclear area, and nucleoprotein components also contributed to HBMEC+HA+HBVP, confirming IR-based phenotyping.

**Figure 5:**
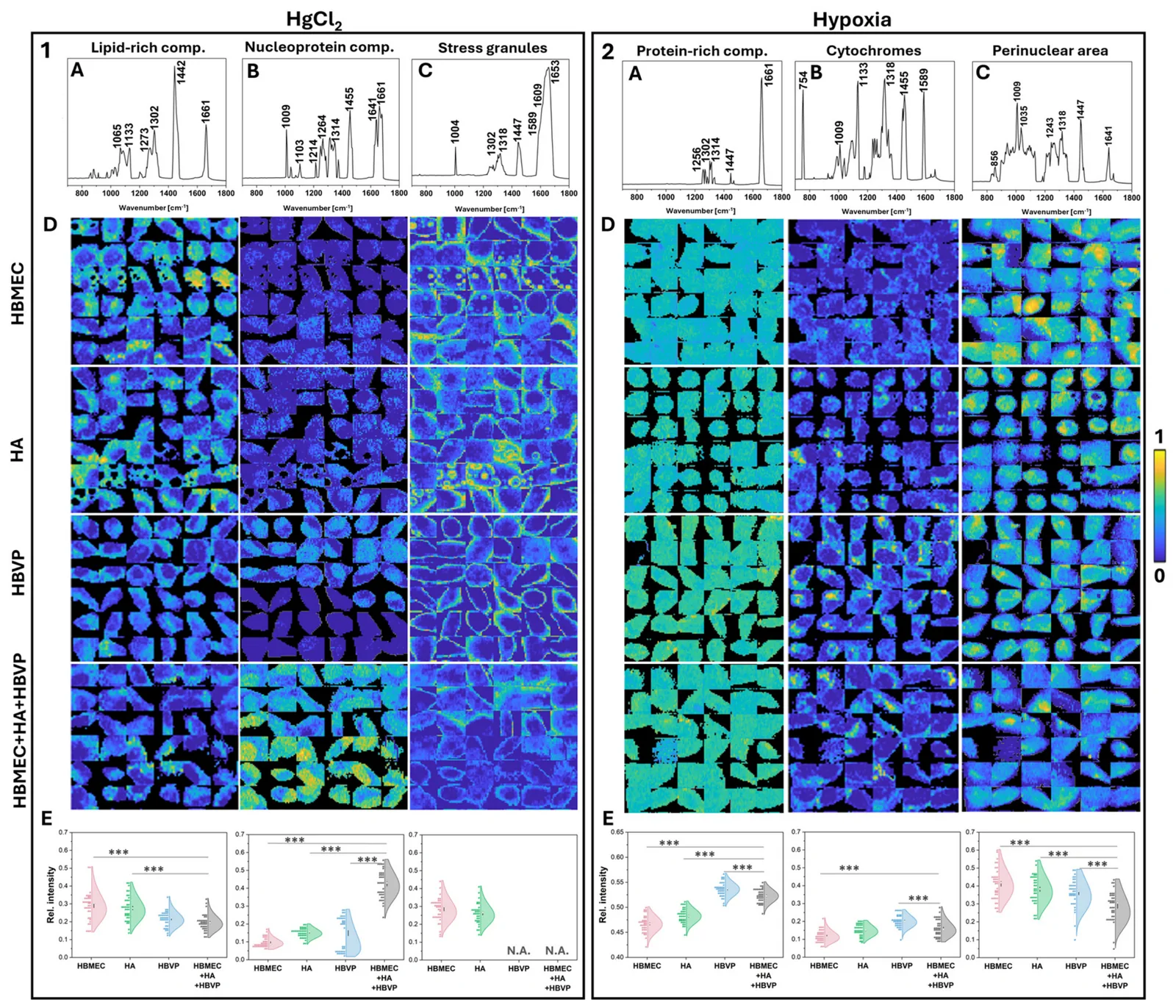
Raman MCR analysis of the NVU cell lines and their triple co-culture under (**1**) HgCl_2_ and (**2**) hypoxic stresses. (**A**–**C**) Selected MCR components and (**D**) their distribution were calculated from representative 36 images in the fingerprint region (500–1800 cm^−1^). All component values in the MCR images were normalized to the (0–1) range. (**E**) Violin plots show the distribution of the median value of component pixels calculated per cell; *** *p* < 0.001; N.A.—not applicable.

HgCl_2_ induced abundant LD formation in HBMEC and HA, in contrast to HBVP, even though LDs are often a stress marker (Figure 5(1A,D), Figures S10 and S11). The number of LD pixels was similar in HBVP and HBMEC+HA+HBVP (Figure 5(1E)). Since the 1660/1440 cm^−1^ signal ratio indicates the unsaturation level of fatty acids, one can observe that this property of LDs did not change due to HgCl_2_ action (Figure 5(1A), Figures S9 and S11) [23]. Interestingly, the nucleoprotein component was particularly prominent in HBMEC+HA+HBVP compared to HBVP, in contrast to HBMEC+HBVP (Figure 5(1B) and Figure S11B). We also identified an MCR component resembling vesicles or vacuoles in HBMEC and HA only (Figure 5(1C) and Figure S11A). Because their Raman spectra resemble those of the cytoplasm and show a predominant protein profile, we assigned them to stress granule-like structures (SGs) (Figure S12). SGs are membraneless organelles formed by liquid–liquid phase separation during cellular stress, including exposure to heavy metals, and Shibuya and co-workers reported their in vitro detection by RS microscopy and fluorescence staining [24].

Under hypoxic conditions, HBVP cellular features contributed to HBMEC+HA+HBVP through the emergence of a highly protein-rich component encompassing the nucleus, the perinuclear region, and the cytoplasm (Figure 5(2A) and Figure S13). Such a phenomenon was not observed in HBMEC+HBVP (Figure S14B). Moreover, an increased number of cytochromes was particularly evident in HBVP and HBMEC+HA+HBVP (Figure 5(2B,D)), revealing similarities between the spectral features of the triple co-culture and those observed in pericyte monocultures. This is also evidenced by the decrease in pixels attributed to the perinuclear component rich in phenylalanine (Figure 5(2C,E) and Figure S14). Notably, under hypoxia, an increase in LD formation was observed distinctly in HBMEC+HA only (Figure S14A).

### 3.6. Spectroscopic Detection of Cell–Cell Contacts

**Figure 6:**
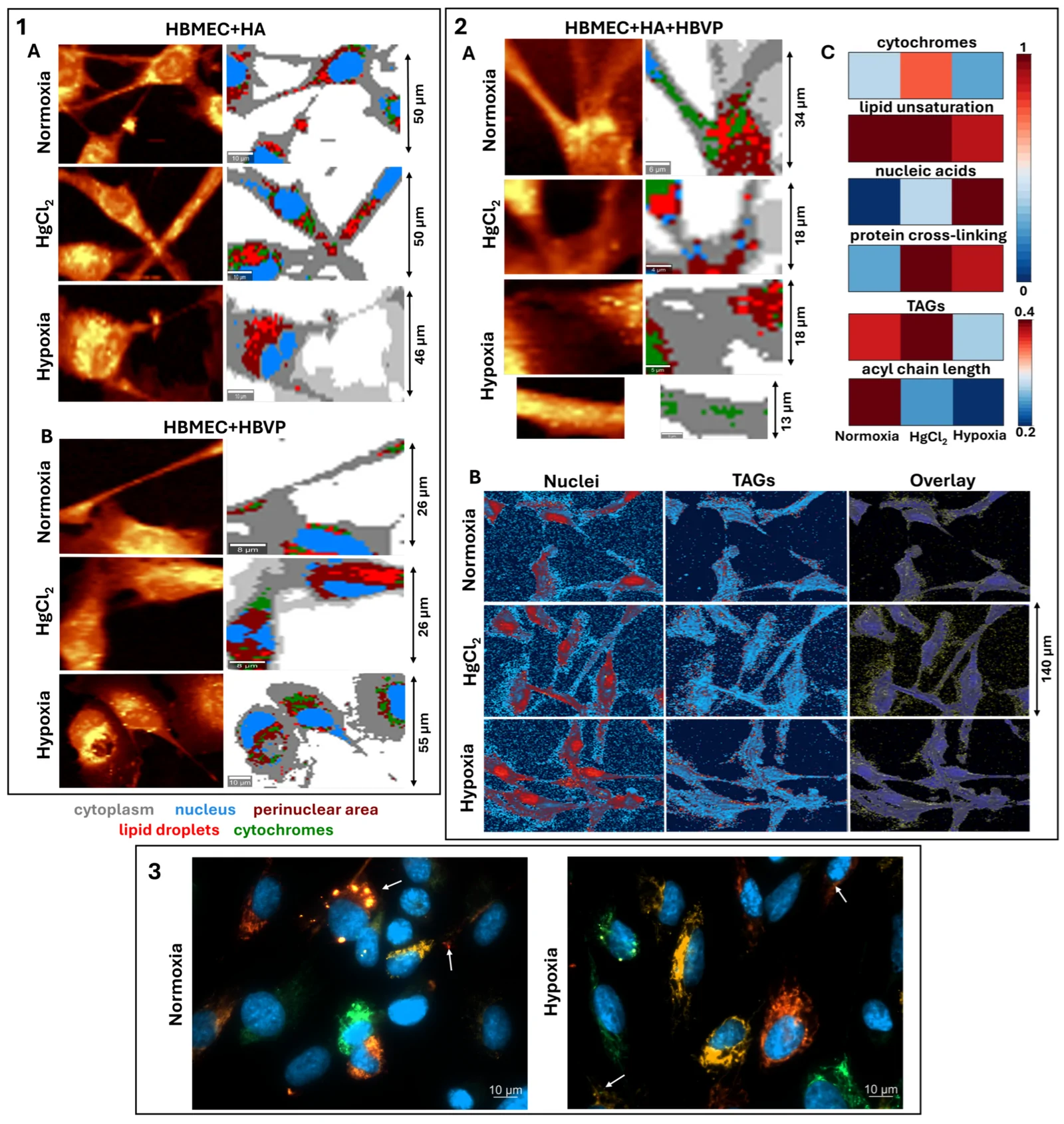
Results of the spectroscopic examination of direct cell–cell interactions in (**1**) di-cultures and (**2**) triculture. Raman image of the cell body (2800–3000 cm^−1^) (left) with the corresponding false-color KMCA map (right) for (**1A**) HBMEC+HA, (**1B**) HBMEC+HBVP, and (**2A**) HBMEC+HA+HBVP. (**2B**) O-PTIR frequency ratio maps for nuclei (ratio of 1240/1740 cm^−1^) and TAG droplets (ratio of 1740/2850 cm^−1^) with their overlay. (**2C**) Heat maps of stress markers calculated from band intensities of cytochromes (RS: ratio of 750/1440 cm^−1^), lipid unsaturation (RS (LDs): ratio of 1660/1440 cm^−1^), protein crosslinking (RS (cytoplasm): ratio of 1040/1005 cm^−1^), nucleic acids (RS (cytoplasm): ratio of 790/1440 cm^−1^), TAGs and acyl chain length in fatty acids (O-PTIR (TAG-rich pixels): ratios of 1740/2850 and 2850/2960 cm^−1^, respectively). The values were normalized to show variation across groups. (**3**) Representative qualitative fluorescence microscopy images of HBMEC+HA+HBVP co-cultures labelled with MitoTracker dyes under normoxic and hypoxic conditions (HA—green, HBMEC—orange, HBVP—red).

Hyperspectral Raman and single-frequency O-PTIR imaging were further used to identify the biochemical composition of contact interfaces between neighboring cells (Figure 6). Raman images constructed to visualize the cell body in the multicellular systems clearly showed how the cells formed the finger-like protrusions called filopodia to establish communication with other cells and make initial contact (Figure 6(1A,B,2A)). Filopodia typically contain actin filaments, as confirmed here by the proteinaceous Raman spectrum (Figure S15.1). KMCA maps revealed that filopodia in normoxia were enriched in small compartments (up to a few microns) containing unsaturated fatty acids and cytochromes (Figure 6(1A,B) and Figure S15.2). Their number decreased regardless of the NVU model or condition. In some cases, spectral clusters associated with nucleic-acid signals were observed outside the nucleus, including within regions of cellular protrusions (Figure 6(2A)) [25]. However, their precise subcellular origin could not be established from the Raman data alone. These contact sites are likely to serve as specialized hubs for intercellular communication and molecular exchange under normal conditions but are inhibited by HgCl_2_ and oxygen depletion. O-PTIR maps at 1740 cm^−1^ for triacylglycerols (TAGs) revealed their accumulation along cell edges and their formation under each condition (Figure 6(2B) and Figure S15.3). A similar observation was found for HBMEC+HA and HBMEC+HBVP. To test whether the cytochrome-rich structures detected by Raman imaging within intercellular contact regions could be associated with mitochondria, HBMEC, HA, and HBVP were separately pre-labelled with distinct MitoTracker dyes before establishment of the triple co-culture (Figure 6(3) and Figure S16). Fluorescence microscopy revealed mitochondria-associated fluorescent signals within regions of close cellular interaction under both normoxic and hypoxic conditions.

Heat maps of IR and Raman signal intensities from structures shared by contacting cells varied across models and stress conditions (Figure 6(2C) and Figure S17). The cytochrome signal was significantly elevated only in HBMEC+HA+HBVP due to HgCl_2_ toxicity. Hypoxia, in turn, slightly reduced it across all models. The most unsaturated fatty acids were observed in HBMEC+HA and HBMEC+HA+HBVP. Hypoxia moderately inhibited their synthesis, except in HBMEC+HBVP (Figure 6(2C) and Figure S17). HgCl_2_ induced fatty acid esterification toward TAGs in HBMEC+HA+HBVP, in contrast to hypoxia (Figure 6(2C)). HBMEC+HA and HBMEC+HBVP responded differently (Figure S17). The stress conditions also led to shortening of the lipid tail in the HBVP-containing models (Figure 6(2C) and Figure S17). Uniquely, HBMEC+HA produced esterified and long acyl chains. Next, the cytoplasmic Raman marker of protein crosslinking and correlated structural alternations was enhanced in the stressed HBMEC+HA+HBVP, which may indicate an increased stiffness of the protein scaffold ([17,20,26], and therein). In addition, elevated nucleic acid signals were observed in this co-culture in response to both stress factors (Figure 6(2C)). The HgCl_2_-exposed HBMEC+HA only exhibited this behavior (Figure S17).

## 4. Discussion

We developed planar 2D neurovascular unit co-culture models to investigate how direct interactions between endothelial cells, astrocytes, and pericytes influence cellular phenotype, cell-cycle regulation, and metabolic responses to stress. In parallel, we established an analytical platform integrating FTIR, Raman, and O-PTIR imaging with multivariate data analysis to characterize cellular phenotypes within interacting cultures and resolve subcellular molecular features. The planar, deliberately sub-confluent configuration provided direct optical access to individual cells and intercellular contact regions, which represented a central requirement of the spectroscopic workflow. Consequently, the endothelial component of the present system does not reproduce the fully confluent and predominantly quiescent endothelial monolayer characteristic of the mature BBB in vivo. This distinction is biologically relevant, because sub-confluent endothelial cells retaining proliferative capacity may respond differently to stress than fully differentiated barrier-forming endothelium. The present model should therefore be interpreted primarily as a platform for studying NVU-associated cellular interactions and molecular stress responses rather than as a direct surrogate of the homeostatic BBB in vivo.

The relevance of this multimodal approach was first demonstrated in phenotyping interacting cells. Classic immunophenotyping showed that endothelial cells constituted the largest cellular fraction in both double and triple co-cultures despite equal initial seeding ratios. After 48 h, HBMEC accounted for approximately 60% of HBMEC+HA cultures and 45% of the triple co-culture, whereas HA represented approximately 40% and 25%, respectively, and HBVP approximately 20–25% of pericyte-containing cultures (Figure 1). These proportions remained relatively stable during stress exposure (Figure S1D–F). However, despite the use of the well-established markers and reproducible gating strategies in flow cytometry-based quantification, the estimated astrocyte proportions should be interpreted cautiously because astrocytes were operationally identified by negative selection (CD31^−^ or CD31^−^CD140b^−^), rather than by a cell-type-specific extracellular marker (Figure 1). Cellular heterogeneity and transitional phenotypes within the co-cultures may additionally generate subpopulations that are not adequately resolved using a limited surface-marker panel.

FTIR-based analyses indicated marked molecular remodeling upon co-culture. These changes may reflect a combination of intercellular interactions, alterations in the cellular microenvironment, and differences in culture conditions. Namely, the cell-specific chemism of each NVU component shown by PCA (Figure 2(1A)) became mixed upon co-culturing (Figure 4B). Multi-class PLS-DA and SVM classification indicated that the spectral features detected in the cell interior of the triple-cell NVU model showed strong similarity to the pericyte reference profile, with partial similarity to the endothelial cell profile (Figure 2(2)). These findings indicate pronounced convergence of spectral features in the multiculture model, reflecting a shift in the overall spectral profile toward the domain associated with the pericyte reference class—not a conversion or loss of the underlying cellular identities. Such a spectral shift may reflect changes in the cellular microenvironment, cell–cell interactions, media effects, and other model-specific factors. Therefore, PLS-DA and SVM classification should be interpreted as reflecting similarity in the measured spectral features and, by itself, does not establish changes in cellular lineage or identity. Our results highlight that routine validation of the cellular composition of BBB and NVU models should be complemented by further multimodal characterization to provide insight into the stability, functionality, and reproducibility of the constituent cell populations, particularly when these models are used for mechanistic and translational studies. These results support the use of multimodal phenotyping alongside conventional immunophenotyping to characterize not only cellular composition but also molecular-state heterogeneity in NVU co-cultures. Importantly, as discussed below, the observed differences between mono- and co-cultures cannot be attributed exclusively to cell–cell interactions because culture medium and substrate conditions also differed between these configurations.

24 h hypoxia did not induce widespread cell death but was associated with reduced cell accumulation and marked alterations in cell-cycle progression (Figure 3). Specifically, the accumulation of endothelial cells and astrocytes in G_2_ was consistent with stress-induced checkpoint engagement and arrest before mitotic entry [27]. The triple co-culture exhibited a more restricted pattern of statistically significant molecular alterations than several mono- and double-culture configurations, indicating that increasing cellular complexity was associated with a distinct pattern of checkpoint regulation rather than uniformly enhanced or suppressed stress signaling. Because endothelial cells in the present sub-confluent model retained proliferative capacity, the stress-induced cell-cycle arrest observed here should be interpreted as a response of the experimental culture rather than as direct evidence of BBB dysfunction in vivo, where brain endothelial cells are predominantly quiescent. Accordingly, these findings may be particularly relevant to stress-associated regulation of proliferative or repair-related cellular states but should not be directly extrapolated to the homeostatic BBB endothelium.

Pericytes showed relatively limited changes in cell number and cell-cycle distribution under hypoxia, consistent with a comparatively stress-resistant phenotype in the present experimental system [28]. In addition, HBMEC+HBVP co-cultures maintained cell number under hypoxia more effectively than HBMEC+HA cultures (Figure S6A,E), while IR phenotyping revealed a shift toward pericyte-like spectral features in pericyte-containing co-cultures (Figure 4B). Together, these observations are consistent with a potential protective contribution of pericytes to the multicellular stress response. However, the present experimental design does not establish causality, because pericyte-specific addition/removal experiments, conditioned-medium studies, or perturbation of candidate protective pathways should be included in further studies. Accordingly, these findings should be interpreted as an association between pericyte presence and a more stress-resistant co-culture phenotype rather than as direct evidence of a pericyte-mediated protective mechanism. In contrast, HBMEC+HA co-cultures exhibited less pronounced preservation of selected cellular parameters under hypoxia than HBMEC+HBVP co-cultures, although these differences do not establish a direct protective role of pericytes or a lack of protective activity of astrocytes. Astrocytes are known for their dual functional roles in ischemia: on the one hand, they deliver neurotrophic factors that protect neurons, but on the other hand, they disrupt tight junctions in the BBB by releasing inflammatory factors and chemokines [29].

At the molecular level, hypoxia produced model-dependent alterations across TP53, p21, p27, pRb, and cyclin–CDK regulators rather than a single uniform checkpoint signature. Hypoxia is known to influence cell-cycle progression through both HIF-dependent and HIF-independent mechanisms, including modulation of cyclins and CDKs [30]. However, the present data do not identify the upstream pathway responsible for the observed arrest. Under hypoxic conditions, limited p53 pathway activity is specific to astrocytes. The arrest of the cycle in the G_2_/M phase, therefore, occurred independently of p53, probably through the activation of the CREB3L1/OASIS pathway and the p21 induction [31]. This result is consistent with work by Li et al., who showed that astrocyte survival under stress, as depicted by the p53 level, is associated with miR-17-5p overexpression [32]. The observed changes provide a basis for selecting specific molecular markers to indicate stress-associated alterations in the NVU co-culture model.

The triple co-culture showed a distinct response to sublethal inorganic mercury exposure, including an increase in inhibitory CDK1 Tyr15 phosphorylation not observed in the corresponding monocultures. Because Tyr15 phosphorylation inhibits CDK1 activity, this finding is consistent with altered regulation of the G_2_/M transition in the multicellular configuration and indicates that multicellular context is associated with altered stress signaling and checkpoint regulation. However, the upstream mechanism responsible for this response cannot be determined from the present dataset. Intercellular and paracrine signaling can modulate cellular stress responses in multicellular BBB systems and may contribute to these configuration-dependent effects [33]. Taken together, astrocytes appear most sensitive to mercury, while pericytes show greater resistance to both stressors. The triple co-culture model exhibited distinct regulatory dynamics, suggesting that cell–cell interactions modulate stress sensitivity and contribute to more physiologically relevant stress responses in vitro. This in vitro observation is consistent with the in vivo study reporting oxidative stress, disrupted mitochondrial function, and activation of apoptotic pathways in neurons and astrocytes upon exposure to inorganic mercury [34]. IR-phenotyping indicated that endothelial cells and astrocytes adopted similar defense mechanisms through interactions, whereas pericytes induced metabolic pathways distinct from those in the individual cell lines.

By MCR mining of Raman images of the single cells, we spatially resolved intra- and inter-cell metabolic markers in the NVU co-culture models. Although the most prominent intracellular features—lipid droplets and cytochromes—were distributed unevenly among the NVU cell lines (Figure 2(4)), both lipid droplet- and cytochrome-associated Raman signals were detected in the investigated NVU co-cultures under normoxic conditions (Figure S9). Spatially resolved imaging further revealed lipid- and cytochrome-associated spectral features within regions of close cellular contact. Notably, mitochondria exhibit dynamic intracellular movement regulated by cytoskeletal elements, primarily microtubules and actin filaments, to be positioned at regions with high metabolic demand [35]. Complementary qualitative MitoTracker imaging identified mitochondria-associated fluorescence signals in these regions (Figure 6(3)). Together, these observations provide morphological and molecular evidence of mitochondria-associated signals at cell–cell interfaces, without establishing intercellular mitochondrial transfer or its underlying mechanism. A high abundance of intracellular mitochondria in pericytes was also characteristic of the NVU models in hypoxia (Figure 5(2B)). However, because the MitoTracker experiment was qualitative and was not designed to quantify mitochondrial exchange or resolve donor–recipient relationships, differences in the frequency or directionality of mitochondria-associated material exchange between normoxic and hypoxic conditions could not be reliably determined here. Alternative mechanisms, including mitochondrial exchange or fusion and the transfer of mitochondria-associated vesicular material, may also contribute to the observed fluorescence patterns [36]. These observations should therefore be regarded as supportive of potential mitochondrial involvement in intercellular contacts rather than as definitive evidence of a specific directional mitochondrial-transfer pathway. In addition, HgCl_2_ induced the formation of nucleoprotein granules in the nuclei of endothelial and pericyte cells in the triple-cell NVU model (Figure 5(1B)). This marker can illustrate conformational transitions in genetic material under severe stress conditions [37]. Inorganic mercury also reduced direct contact and mitochondria-associated signals within intercellular contact regions (Figure 6(1,2)). The observed stress-associated molecular features further highlight the heterogeneous responses of individual NVU cell types and the triple co-culture to HgCl_2_ exposure, without establishing a specific protective role of pericytes. Hypoxia and inorganic mercury also triggered substantial cytoplasmic lipid and protein remodeling in the triple-cell NVU model, including changes in lipid unsaturation, fatty acid esterification, and acyl-chain length that are consistent with lipid remodeling associated with oxidative stress and lipid peroxidation, together with protein crosslinking probably associated with increased protein scaffold stiffness. Under these conditions, structural modifications in key morphological elements of the NVU cells affected cell mobility, membrane integrity, and permeability (Figure 5(2C)). However, whether these molecular alterations affect cell mobility, membrane integrity, or permeability remains an open question that warrants further investigation.

An important limitation of the present model is that it does not permit direct assessment of endothelial barrier tightness by TEER or transendothelial tracer permeability. These approaches conventionally require a confluent endothelial monolayer formed on a permeable support that separates luminal and abluminal compartments [2,38]. In the present system, all cell types were directly co-cultured on the same planar surface and intentionally maintained below full confluence to enable single-cell and subcellular spectroscopic imaging. Thus, the model was designed to investigate cellular cross-talk, metabolic remodeling, and stress responses rather than physical barrier tightness. Future studies combining the present spectroscopic approach with complementary barrier-forming systems may help relate the molecular signatures identified here to quantitative changes in BBB permeability and resistance.

Another limitation is differences in culture conditions between monoculture and co-culture configurations. Co-cultures were maintained in mixed media and seeded on fibronectin-coated surfaces, whereas the corresponding monocultures were maintained in cell-type-specific media and, for HA and HBVP, on poly-L-lysine-coated surfaces. Future experimental designs should include dedicated monoculture controls that replicate the complete co-culture medium and substrate conditions. Despite this issue, we compared stressed samples with matched controls under the same medium and substrate conditions within each culture configuration. This allowed us to assess stress-induced effects independently in each model.

## 5. Conclusions

In summary, we characterized NVU in vitro models comprising endothelial cells, astrocytes, and pericytes in double- and triple-cell configurations to investigate cellular and molecular responses to sustained hypoxia and sublethal heavy-metal neurotoxic stress. Different culture configurations were associated with distinct patterns of cell-cycle regulation and metabolic remodeling. Under the stress conditions investigated, the triple co-culture showed a comparatively limited pattern of cell-cycle-related alterations. Pericyte-containing cultures were associated with greater preservation of selected cellular parameters under hypoxia; however, these observations do not establish a causal protective mechanism. Moreover, differences between monocultures and co-cultures cannot be attributed exclusively to cell–cell interactions, as culture medium and substrate conditions also varied.

Integrating molecular bioassays with FTIR, Raman, and O-PTIR chemical imaging provides a complementary approach for label-free characterization of biochemical heterogeneity at the whole-cell, subcellular, and intercellular-contact levels. Analysis of hyperspectral data enabled the identification of intracellular regions with distinct molecular signatures and revealed alterations in lipid-, protein-, cytochrome-, and nucleic-acid-associated signals, while qualitative fluorescence imaging identified mitochondria-associated signals in regions of close cellular interaction. These observations do not establish intercellular mitochondrial transfer. Overall, the integration of targeted molecular analyses with spatially resolved chemical imaging provides a valuable framework for characterizing cellular phenotypes and stress-associated molecular changes in multicellular NVU models.

## Data Availability

The datasets generated and/or analyzed during the current study are available in the RODBUK repository, https://doi.org/10.57903/UJ/9WFFCU.

## Supplementary Materials

The following supporting information can be downloaded at https://www.mdpi.com/article/10.3390/cells15201873/s1: Supplementary Materials and Methods, and Supplementary Results [39].

## Abbreviations

The following abbreviations are used in this manuscript:

BBB	Blood–Brain Barrier
BCRP	Breast Cancer Resistance Protein
BMECs	Brain Microvascular Endothelial Cells
CNS	Central Nervous System
FPA	Focal Plane Array
FTIR	Fourier Transform Infrared
GFAP	Glial Fibrillary Acidic Protein
GLUT1	Glucose Transporter Type 1
HA	Human Astrocytes
HBVP	Human Brain Vascular Pericytes
HBMEC	Human Brain Microvascular Endothelial Cells
iPSCs	Human-induced Pluripotent Stem Cells
KMCA	K-means Cluster Analysis
LDs	Lipid droplets
LV	Latent Variable
MCR	Multivariate Curve Resolution
ML	Machine Learning
MRP1	Multidrug Resistance-Associated Protein 1
NVU	Neurovascular unit
O-PTIR	Optical Photothermal Infrared spectroscopy
PCA	Principal Component Analysis
PDGFR-β	Platelet-Derived Growth Factor Receptor-β
P-gp	permeability glycoprotein
PLS-DA	Partial Least Squares Discriminant Analysis
ROI	Region of interest
SGs	Stress granule-like structures
SVM	Support Vector Machine
TAGs	Triacylglycerols
TEER	Transendothelial Electrical Resistance
ZO-1	Zonula occludens-1
