# Supplementary material for: Decoding Cell–Cell Interactions in Neurovascular Unit Co-Cultures Through Molecular Vibrational Spectroscopy and Biology
Source: Cells. 2026 Oct 15;15(20):1873. doi: 10.3390/cells15201873 (PMC13702744; doi:10.3390/cells15201873)
Supplement: Supplementary file 1 [file cells-15-01873-s001.zip › Interaction_Supplementary_Materials_and_Methods.pdf]

Supplementary Information
# – Materials and Methods

Decoding Cell–Cell Interactions in Neurovascular Unit Co-Cultures through Molecular Vibrational Spectroscopy and Biology

Aleksandra Pragnąca[1,2], Agata Borkowska[3], Anna Antolak[1], Monika Leśniak[3], Karolina Augustyniak[1], Iga Czechowska[1], Kamilla Malek[1], Robert Zdanowski[3]

[1] Jagiellonian University in Krakow, Faculty of Chemistry, Department of Chemical Physics, Gronostajowa 2, 30-387, Krakow, Poland

[2] Jagiellonian University in Krakow, Doctoral School of Exact and Natural Sciences, prof. S. Lojasiewicza 11, 30-348 Krakow, Poland

[3] Military Institute of Medicine National Research Institute, Laboratory of Molecular Oncology and Innovative Therapies, Szaserow 128, 04-141 Warsaw, Poland

MATERIALS AND METHODS 2

Cell Culture 2

Stress Induction - Hypoxia 3

Stress Induction - $HgCl_2$ Dose Selection and Rationale 4

RNA Isolation and RT-qPCR 5

Western Blotting 6

Flow Cytometry - Immunophenotyping 9

Apoptosis Assay 10

Cell Cycle Analysis 10

Live Fluorescence Staining of Mitochondria 11

Statistical Analysis of biological data 12

Preprocessing and analysis of Raman, FTIR, and O-PTIR data 13

SUPPORTING TABLES 16

MATERIALS AND METHODS

Cell Culture

Immortalized human brain microvascular endothelial cells (HBMEC; Innoprot, Derio (Bizkaia), Spain), as well as primary astrocytes, and pericytes (HA and HBVP, respectively; ScienCell, Carlsbad, USA) were cultured at 37 °C, 5% CO_2. HBMECs were grown in Endothelial Cell Medium (ScienCell, Carlsbad, CA, USA), HA in Astrocyte Medium (ScienCell, Carlsbad, CA, USA), and HBVP in Pericyte Medium (ScienCell, Carlsbad, CA, USA), each supplemented per the supplier's protocols (ScienCell, Carlsbad, USA). For monocultures, surface coatings were applied in accordance with the respective manufacturers' recommendations: HBMEC were seeded on fibronectin-coated dishes (Thermo Fisher Scientific, Carlsbad, CA, USA), while HA and HBVP were seeded on poly-L-lysine-coated surfaces (MilliporeSigma, Burlington, MA, USA). Cells were used at passage 5th and seeded at 10,000 cells/cm^2, a density maintained below full confluency to meet the optical requirements of FTIR and Raman spectroscopic imaging.

Co-cultures were established as HBMEC+HA (1:1), HBMEC+HBVP (1:1), and HBMEC+HA+HBVP (1:1:1). Each cell type was maintained separately in its dedicated monoculture medium until sufficient cell numbers were obtained, at which point all cell lines were simultaneously harvested by enzymatic detachment and combined in a single suspension prior to seeding. Co-cultures were seeded onto fibronectin-coated vessels shared by all cell types in the given configuration. The mixed culture medium was composed of the dedicated monoculture media for each cell type, combined in proportions matching the seeding ratio - that is, equal volumes for 1:1 double co-cultures and equal thirds for the 1:1:1 triple co-culture. Cells were allowed to establish co-culture interactions over a total experimental period of 48 hours. Medium was not replaced during this period except at the point of stressor application (24 h after seeding), when cultures received either hypoxia-conditioned medium, medium supplemented with 20 µM $HgCl_2$, or matched normoxic control medium, and were subsequently maintained for a further 24 h under the respective stress or control conditions. Hematopoietic contamination was excluded by the lack of CD45 expression (**Fig. M1**).

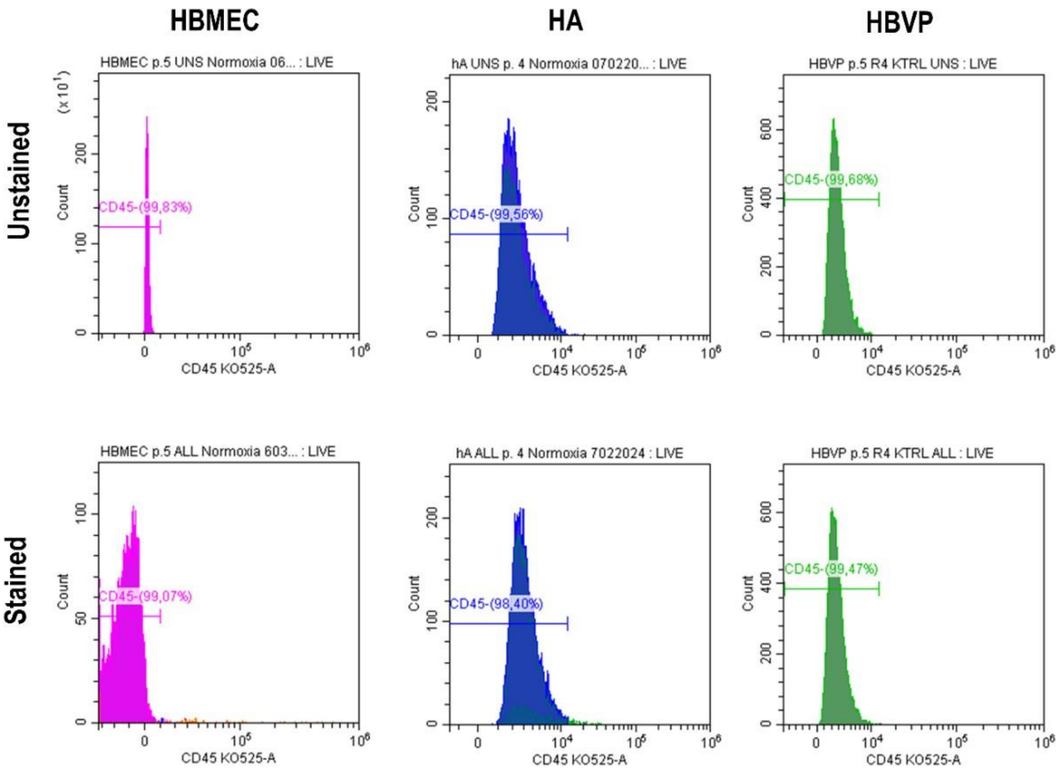

Figure M1. Representative flow-cytometry histograms of CD45 expression in unstained (top row) and CD45-stained (bottom row) HBMEC, HA, and HBVP monocultures. Data shown are representative of n≥3 experiments.

Stress Induction - Hypoxia

For hypoxic stress, cells were transferred to a calibrated hypoxia workstation (BioSpherix C-Chamber, BioSpherix, Parish, NY, USA) maintained at 1% O_2, 5% CO_2, and 37 °C for 24 hours. Oxygen concentration within the chamber was continuously monitored throughout the experiment by the integrated ProOx sensor system; a representative pO_2 log from a 24-hour experimental run is provided in **Figure M2.** As shown, pO_2 remained stable at the target level of approximately 1% O_2 throughout the exposure period. The transient spikes in pO_2 observed in the log (reaching up to ~1.9%) correspond to brief atmospheric oxygen ingress during necessary chamber openings — for example, during medium exchange or sample handling - after which the workstation rapidly restored the target oxygen concentration within minutes. The set point within the working chamber was maintained at 2% O_2 to account for oxygen consumption by the cells and to ensure that the actual pO_2 experienced by the cultures remained consistently at or below 1% pO_2 These data confirm that biologically meaningful hypoxic conditions were maintained for the full duration of the experiment, with only transient and rapidly corrected deviations.

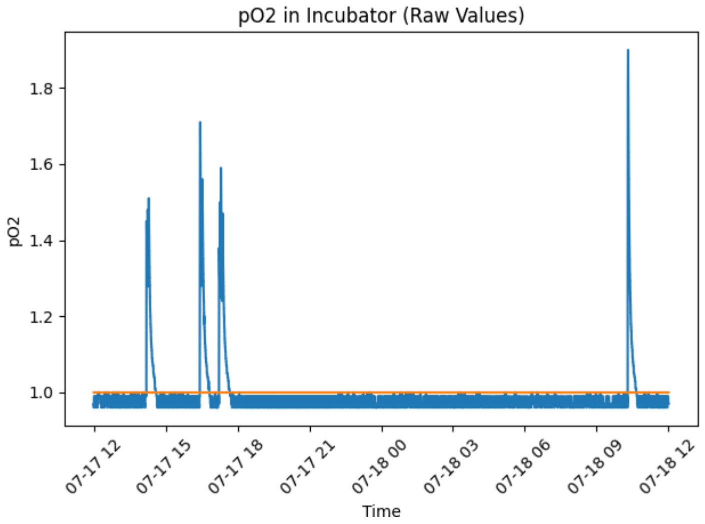

Figure M2. Representative O_2 partial pressure recording from a 24-hour incubation in a BioSpherix chamber. The blue line represents the process value, and the orange line represents the set point.

The 24-hour exposure time point was selected to capture the cellular adaptive response to sustained hypoxia — the phase of cell-cycle and metabolic reprogramming - rather than the transient HIF-1α accumulation that characterises the acute response (0–8 h) and which undergoes negative feedback-mediated decline under prolonged exposure. Controls were maintained under normoxic conditions (21% O_2) and received matched conditioned medium prepared under the same atmospheric conditions.

Stress Induction - $HgCl_2$ Dose Selection and Rationale

Inorganic mercury ($HgCl_2$) was selected as a model neurotoxicant due to its well-documented ability to impair BBB integrity through multiple mechanisms, including disruption of tight junction proteins, induction of oxidative stress, and mitochondrial dysfunction, without requiring metabolic activation - unlike organic mercury species. To determine a biologically relevant, sublethal concentration for subsequent experiments, a dose-response assessment was performed using the Alamar Blue (resazurin) metabolic activity assay. HBMECs were selected for dose optimisation as the only rapidly proliferating cell line in the panel and the component most likely to exhibit the earliest and most quantifiable metabolic response. HBMECs were exposed to four concentrations of $HgCl_2$ - 10, 20, 50, and 100 µM - for 24 hours under normoxic conditions, alongside an untreated control. After that, cells were incubated with Alamar for 4 h. After that time fluorescence was measured using 560/590 nm (excitation/emission) filter settings via a Varioscan LUX microplate reader (Thermo Fisher Scientific, Waltham, MA, USA).

At 50 µM and 100 µM, $HgCl_2$ induced pronounced cytotoxicity, with marked loss of cell viability observed across replicate experiments, rendering these concentrations unsuitable for the study of sublethal stress responses. At 10 and 20 µM, a non-significant trend towards increased Alamar Blue signal was observed relative to the untreated control (**Fig. M3**), which we interpret as a metabolic stress response - consistent with mitochondrial perturbation and compensatory metabolic activation - rather than reflecting an increase in cell number or viability per se. The concentration of 20 µM $HgCl_2$ was selected for all subsequent experiments as an intermediate dose situated between the initial metabolic perturbation observed at 10 µM and the pronounced cytotoxicity evident at 50 µM, thereby enabling the study of sublethal stress responses - including cell-cycle alterations and metabolic reprogramming - without confounding cell death. Mercury exposure was performed in normoxic conditioned medium for all experimental groups.

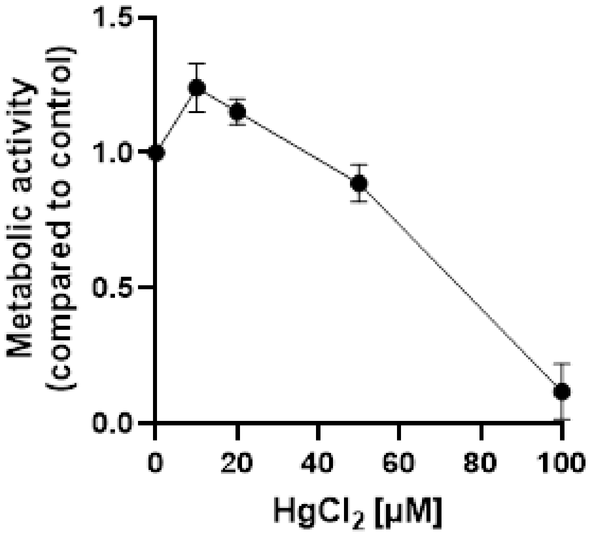

Figure M3. Dose-dependent effect of $HgCl_2$ on HBMEC metabolic activity assessed by the Alamar Blue assay. Data are presented as mean ± SEM ($n \geq 4$ independent experiments).

RNA Isolation and RT-qPCR

Total RNA was extracted using the Direct-zol™ RNA Miniprep Kit (Zymo Research, Irvine, CA, USA) according to the manufacturer's protocol. Briefly, cells were lysed directly in TRIzol reagent (Thermo Fisher Scientific, Carlsbad, CA, USA), kept overnight in -80°C, then the probes were thawed on ice, and the lysate was processed through the silica column. RNA was eluted in nuclease-free water and quantified using a Varioscan LUX microplate reader

(Thermo Fisher Scientific, Waltham, MA, USA). RNA purity was assessed by the A_{260}/A_{280} ratio; samples with $A_{260}/A_{280} \geq 1.8$ were considered acceptable for downstream applications.

Single-stranded cDNA was synthesised from 1 - 1.5 µg of total RNA in a 20 µL reaction using the High-Capacity cDNA Reverse Transcription Kit (Applied Biosystems, Cat. No. 4368814; Thermo Fisher Scientific, Carlsbad, CA, USA) according to the manufacturer's protocol. The kit uses random hexamer primers and MultiScribe™ Reverse Transcriptase (50 U/µL) for quantitative conversion of all RNA species. Thermal cycling conditions for reverse transcription were as follows: 25 °C for 10 min (primer annealing), 37 °C for 120 min (cDNA synthesis), 85 °C for 5 min (enzyme inactivation), and hold at 4 °C. The resulting cDNA was diluted 1:5 in nuclease-free water prior to qPCR and stored at -20 °C until use.

Quantitative PCR was performed using PowerUp™ SYBR™ Green Master Mix (Applied Biosystems, Cat. No. A25741; Thermo Fisher Scientific, Carlsbad, CA, USA) on a CFX Connect Real-Time PCR Detection System (Bio-Rad Laboratories, Hercules, CA, USA). Each 10 µL reaction contained 5 µL of 2× PowerUp SYBR Green Master Mix, 300-500 nM of each primer (forward and reverse), and cDNA template. Standard cycling conditions were applied: initial UDG activation at 50 °C for 2 min, followed by Dual-Lock™ Taq polymerase activation at 95 °C for 2 min, then 40 cycles of denaturation at 95 °C for 15 s and combined annealing/extension at 60 °C for 1 min. A melt curve analysis (65-95 °C, 0.5 °C increments) was performed at the end of each run to confirm amplicon specificity and the absence of primer-dimer formation or non-specific products. All reactions were run in technical duplicates, and no-template controls (NTC) were included on every plate.

The following genes were analysed: TP53, RB1, RBL1, CDKN1B, CCND1, CCNE2, CCNA2, CDK1, and CCNB1. GAPDH served as the reference gene for normalisation. Primer sequences are provided in **Table M1**. Relative gene expression was calculated using the $2^{-\Delta\Delta Ct}$ method, $\log_2$-transformed, and expressed relative to the normoxic untreated control.

Western Blotting

Proteins were extracted in RIPA buffer supplemented with protease and phosphatase inhibitors to preserve phosphoprotein integrity and quantified using the BCA Protein Assay Kit (Thermo Fisher Scientific, Carlsbad, CA, USA). Lysates were vortexed, stored at -80 °C overnight, thawed on ice for 30 min, and vortexed again, followed by clarification by centrifugation at 14,000 × g for 10 min at 4 °C. Total protein concentration in the supernatant

was determined according to the manufacturer's protocol, using bovine serum albumin (BSA) as the standard.

Equal amounts of protein (20 µg per lane) were denatured in Laemmli sample buffer at 95 °C for 5 min, separated by SDS-PAGE on 4-20% precast polyacrylamide gradient gels (Mini-PROTEAN TGX, Bio-Rad Laboratories, Hercules, CA, USA) under reducing conditions, and electrophoretically transferred to PVDF membranes (Bio-Rad Laboratories, Hercules, CA, USA) using a wet transfer system in TG transfer buffer (25 mM Tris, 192 mM glycine, 20% methanol). Membranes were pre-equilibrated in transfer buffer prior to transfer. Transfer efficiency and equal loading were verified by Ponceau S staining.

Membranes were blocked for 1 h at room temperature with 5% non-fat dry milk in Tris-buffered saline containing 0.1% Tween-20 (TBST) to reduce non-specific binding. Membranes were then incubated overnight at 4 °C on a rocking platform with primary antibodies against Cyclin D1, phospho-retinoblastoma protein (pRb), p27 (CDKN1B), p21 (CDKN1A), Cyclin A2, Cyclin B1, phospho-cdc2 (CDK1 Tyr15), GFAP, and GAPDH (loading control). Full details of antibody clones, catalogue numbers, dilutions, host species, and manufacturers are provided in **Table M2**.

Following primary antibody incubation, membranes were washed three times in TBST (10 min each) and incubated for 1 h at room temperature with HRP-conjugated secondary antibodies (Vector Laboratories, Newark, CA, USA). Membranes were washed again three times in TBST prior to detection.

A chemiluminescent signal was developed using the ECL Western Blotting Detection Reagent (Santa Cruz Biotechnology, Dallas, TX, USA) according to the manufacturer's instructions and captured using the Alliance Q9 chemiluminescence imaging system (Uvitec, Cambridge, UK). Band intensities were quantified using ImageJ software (National Institutes of Health, Bethesda, MD, USA) and normalised to GAPDH within each membrane.

Full-length, uncropped Western blot images corresponding to the cropped GFAP and GAPDH bands shown in the manuscript are provided in Figure M3, together with the relevant molecular-weight markers and sample identities. These blots were included for qualitative characterization of GFAP expression and were not used for densitometric analysis.

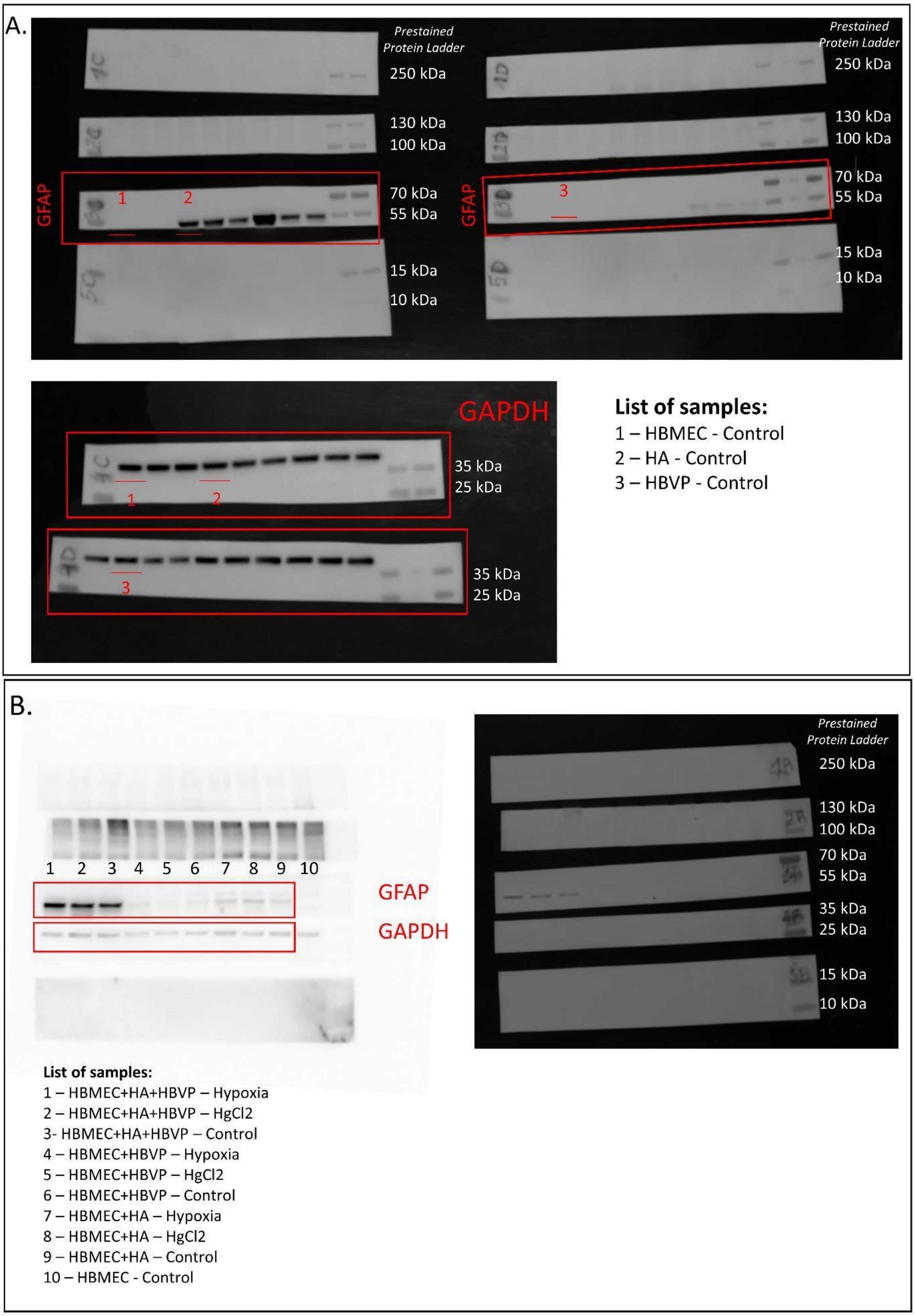

Figure M4. Full-length uncropped Western blot images for GFAP and GAPDH.

Full-length chemiluminescence images corresponding to the cropped GFAP and GAPDH bands presented in the manuscript. Molecular-weight markers and sample identities are indicated. Panel A shows HBMEC, HA, and HBVP monocultures under control conditions. Panel B shows the indicated mono- and co-culture configurations under control, hypoxic, and $HgCl_2$-treated conditions. Red boxes indicate the regions used for presentation in the cropped figures.

Flow Cytometry - Immunophenotyping

Cells were detached using Accutase solution (Thermo Fisher Scientific, Carlsbad, CA, USA) for 5-10 min at 37 °C, a mild enzymatic dissociation reagent selected to preserve surface antigen integrity. Following detachment, cells were washed twice in phosphate-buffered saline (PBS) supplemented with 2% fetal bovine serum (FACS buffer) and centrifuged at 300 × g for 5 min to remove residual Accutase. Cell counts and viability were assessed prior to staining.

Approximately 1-5 × 10^5 cells per sample were resuspended in 100 µL FACS buffer and incubated with fluorophore-conjugated antibodies targeting the following surface antigens: CD31 (endothelial marker), CD140b/PDGFRβ (pericyte marker), CD105/Endoglin (endothelial co-receptor), CD45 (hematopoietic contamination marker) and P-gp/ABCB1 (drug efflux transporter) . Incubation was performed at room temperature for 30 min in the dark with gentle agitation. No prior blocking step was applied, as the FACS buffer containing serum was used throughout to minimize non-specific binding. Full details of antibody clones, fluorochrome conjugates, catalogue numbers, manufacturers, and concentrations used are provided in **Table M3**. Following surface staining, all samples were washed once in FACS buffer and fixed in 4% formaldehyde for 15 min at room temperature. Fixed cells were washed twice in PBS and resuspended in an appropriate volume for acquisition.

Dead cells and debris were excluded during data acquisition based on light scatter characteristics (forward scatter area vs. side scatter area; FSC-A vs. SSC-A) rather than viability dye staining, given that cells were fixed prior to analysis. $CD45^+$ events were additionally excluded from all analyses to eliminate any residual hematopoietic contamination. A minimum of 10,000 events per sample were acquired on a CytoFLEX flow cytometer (Beckman Coulter, Brea, CA, USA). Compensation controls (single-stained samples and fluorescence-minus-one controls where applicable) were prepared for each fluorochrome used. Data were analysed using CytExpert software v.2.4 (Beckman Coulter).

Apoptosis Assay

Apoptosis and cell death were assessed using the FITC Annexin V/Propidium Iodide (PI) Apoptosis Detection Kit (BD Pharmingen, San Jose, CA, USA). To ensure complete capture of the dying cell population - including both adherent and detached cells, the latter of which are enriched for late apoptotic and necrotic events - the floating cell fraction was collected from the conditioned medium by centrifugation at 300 × g for 5 min and pooled with the adherent fraction harvested by Accutase-mediated detachment. The combined cell suspension was washed twice in ice-cold PBS and once in 1× Annexin V Binding Buffer (10 mM HEPES pH 7.4, 140 mM NaCl, 2.5 mM $CaCl_2$) provided in the kit.

Cells were resuspended in 100 µL of 1× Annexin V Binding Buffer at a concentration of approximately 1×10^6 cells/mL. FITC-conjugated Annexin V (5 µL) and PI solution (5 µL) were added simultaneously, and the samples were incubated for 30 min on ice in the dark to allow optimal binding of Annexin V to phosphatidylserine residues externalised on the outer leaflet of the plasma membrane. Following incubation, 400 µL of Annexin V Binding Buffer was added to each sample without washing, and samples were immediately analysed by flow cytometry to prevent redistribution of the apoptotic signal.

Samples were acquired on a CytoFLEX flow cytometer (Beckman Coulter, Brea, CA, USA) using FITC (Annexin V) (Excitation: 488 nm, Fluorescence Channel: 525/540 BP) and PE or PerCP (PI) (Excitation: 488 nm, Fluorescence Channel: 585/42 BP) detection channels. A minimum of 10,000 events per sample were recorded. Compensation was performed using single-stained controls (Annexin V only and PI only), and an unstained control was included in each experimental run to set baseline scatter and fluorescence gates. Four cell populations were identified based on Annexin V and PI signal: viable cells (Annexin V^-/PI^-), early apoptotic cells (Annexin V^+/PI^-), late apoptotic cells (Annexin V^+/PI^+), and necrotic cells (Annexin V^-/PI^+). Data were analysed using CytExpert software (Beckman Coulter).

Cell Cycle Analysis

Cell cycle distribution was determined by propidium iodide (PI)-based DNA content analysis using the Tali™ Cell Cycle Kit (Thermo Fisher Scientific, Carlsbad, CA, USA). Cells were harvested by Accutase-mediated detachment, washed once in PBS, and counted to ensure consistent cell numbers across samples. Approximately 1×10^6 cells per sample were pelleted by centrifugation at 300 × g for 5 min, the supernatant was aspirated, and the pellet was resuspended in 500 µl of PBS and fixed by dropwise addition of ice-cold 70% ethanol while

vortexing gently, to minimise cell clumping. Fixed cells were stored overnight at -20 °C to ensure complete permeabilisation of the cell membrane and allow full ethanol penetration.

Prior to staining, ethanol was removed by centrifugation at 300 × g for 5 min, and cells were washed twice in PBS to eliminate residual ethanol, which can interfere with PI intercalation. Cell pellets were resuspended in the PI-based staining reagent provided in the Tali™ Cell Cycle Kit according to the manufacturer's protocol and incubated for 30 min at room temperature in the dark to allow stoichiometric intercalation of PI into double-stranded DNA.

Stained samples were acquired on a CytoFLEX flow cytometer (Beckman Coulter, Brea, CA, USA) using the PE detection channel (excitation 488 nm, Fluorescence Channels: 585/42 BP). Singlets were gated using the PI-area vs. PI-width plot (FSC-A vs. FSC-H) to exclude cell doublets and aggregates, which would otherwise artificially inflate the G_2/M fraction. A minimum of 10,000 singlet events per sample were acquired. DNA content histograms were exported and analysed in FlowJo software v.10 (BD Life Sciences, Ashland, OR, USA) using the Dean-Jett-Fox mathematical model for cell cycle fitting, which accounts for S-phase broadening and provides robust estimation of G_1, S, and G_2/M fractions.

Live Fluorescence Staining of Mitochondria

To visualise mitochondrial distribution and assess intercellular mitochondrial transfer in triple co-culture, each cell type was labelled with a spectrally distinct, mitochondria-selective MitoTracker dye (Thermo Fisher Scientific, Carlsbad, CA, USA) prior to co-culture establishment. MitoTracker CMTMRos and FM dyes differ in their retention mechanism: CMTMRos (used for HBMEC) is retained after fixation due to covalent binding to mitochondrial proteins via thiol-reactive chloromethyl groups, whereas FM dyes (used for HA and HBVP) are retained through membrane potential-independent sequestration following aldehyde fixation. This dye selection ensured that the mitochondrial signal was preserved throughout the fixation step and remained attributable to the cell type of origin.

Upon reaching 80-90% confluency in monoculture, HBMEC, HA, and HBVP were incubated with 150 nM MitoTracker Orange CMTMRos, 200 nM MitoTracker Green FM, and 200 nM MitoTracker Red FM, respectively. Each dye was diluted in the dedicated culture medium appropriate for the respective cell type to avoid osmotic stress or medium-incompatibility artefacts. Cells were incubated at 37 °C, 5% CO_2 for 1 hour to allow complete mitochondrial uptake and sequestration of the dye. Following staining, cells were washed once with pre-warmed PBS to remove unincorporated dye, harvested by Accutase-mediated detachment,

and counted. All three labelled cell populations were then combined in a 1:1:1 ratio and co-seeded onto fibronectin-coated Chamber Slides (Thermo Fisher Scientific, Carlsbad, CA, USA) at a total density of 10,000 cells/cm^2.

Immediately after seeding, half of the culture vessels were transferred to the hypoxia workstation (1% O_2, 5% CO_2, 37 °C; BioSpherix, Parish, NY, USA), while the remaining vessels were maintained under normoxic conditions (21% O_2). After 24 hours of co-culture, nuclei were counterstained with Hoechst 33342 (bisbenzimide H33342 trichloride; Sigma-Aldrich, Burlington, MA, USA) at a concentration of 1 µg/mL in PBS for 15 min at 37 °C. Cells were subsequently fixed with 4% paraformaldehyde (PFA) in PBS for 15 min at 37 °C, washed gently with PBS, and slides were sealed with Immunofluorescence Mounting Medium (Dako, Glostrup, Denmark) and stored at 4 °C in the dark until imaging.

Fluorescence images were acquired on a Zeiss Axio Observer 7 inverted microscope (Zeiss, Oberkochen, Germany) equipped with a 63× oil immersion objective (NA 1.4). Multi-channel acquisition was performed sequentially across the following channels: DAPI/Hoechst (excitation ~405 nm, nuclei), FITC/GFP (excitation ~488 nm, MitoTracker Green FM - HA), Cy3/TRITC (excitation ~561 nm, MitoTracker Orange CMTMRos - HBMEC), and Cy5 (excitation ~633 nm, MitoTracker Red FM — HBVP). Sequential acquisition mode was used to minimise spectral bleed-through between channels. Images were analysed using ZEN Blue v. 3.4 software (Zeiss). Potential mitochondrial transfer events were identified qualitatively by visual inspection as the presence of a MitoTracker signal from one cell type within the cytoplasm of a morphologically distinct neighbouring cell type, based on cell body size, shape, and nuclear characteristics. Negative controls consisting of unlabelled cells processed in parallel under identical conditions were included in each experimental run to confirm dye specificity, establish channel baseline fluorescence, and exclude autofluorescence or non-specific signal contributions.

Statistical Analysis of biological data

Biological data were analysed using GraphPad Prism version 10.4.1 (GraphPad Software, San Diego, CA, USA). For cellular assays, Control, $HgCl_2$, and hypoxia conditions originating from the same independent biological experiments were treated as matched observations. Data were analysed separately for each cellular model using one-way repeated-measures ANOVA followed by Dunnett's multiple-comparisons test, with the Control condition used as the reference. The predefined comparisons were Control vs $HgCl_2$ and Control vs Hypoxia.

RT-qPCR data were analysed using ordinary two-way ANOVA, with treatment condition (Control, $HgCl_2$, and Hypoxia) and cellular model as the two experimental factors. The full model included the main effects of treatment and cellular model as well as the treatment × cellular model interaction. Multiple comparisons were performed using Tukey's multiple-comparisons test.

Western blot densitometry was analysed separately for each protein and cellular model using two-tailed paired Student's t-tests, as Control and stress-treated samples originated from the same independent biological experiments. Western blot analyses were based on three independent biological experiments ($n = 3$). No formal multiple-comparison correction was applied to these predefined pairwise Western blot comparisons.

No individual biological replicate values were selectively excluded from the analyses. Statistical significance was defined as $p \leq 0.05$; where Dunnett's or Tukey's multiple-comparisons procedures were applied, adjusted p-values were used. For all biological assays, n represents the number of independent biological experiments rather than technical replicates, and exact n values are reported in the corresponding figure legends. Unless otherwise stated, data are presented as mean ± SEM together with individual biological replicate values.

Preprocessing and analysis of Raman, FTIR, and O-PTIR data

Spectral data sets were analyzed using a combination of software packages, including WITec Project (version 5.0, WITec, Germany), CytoSpec (version 2.00.01), MatLab (R2020a, MathWorks, Natick, USA), PLS_Toolbox (version 9.2.1, Eigenvector Research, Manson, USA), OPUS (version 7.2, Bruker, Billercia, USA), and Origin 9.1 (version 2020b, OriginLab Corporation, Northampton, USA).

Pre-processing of Raman images involved applying a cosmic-ray removal filter with a size of 3 and a dynamic factor of 8, followed by baseline correction using a third-order polynomial.

Raman spectral data analysis was performed on monocultured and co-cultured cell maps using Multivariate Curve Resolution (MCR). Spectral preprocessing, including SNV (Standard Normal Variate) normalization and truncation to 700-1800 cm^{-1}. We compiled and analyzed datasets comprising 36 Raman images per cell type using an unconstrained MCR model with seven initial components. After removing the background component, the model was recalculated to generate spatial distribution maps and spectral profiles for the identified components. Finally, we selected seven components to capture the main cellular and subcellular spectral contributions, including lipid droplets, the nucleus, a cytochrome-rich class,

the perinuclear region, and stress granules. Increasing the number of components did not identify additional biologically meaningful classes. The images were converted to 8-bit format to standardize the intensity range (ImageJ). A threshold was applied to generate a mask highlighting the most intense regions within the cells. Using this mask, the percentage of the area occupied by these regions was automatically calculated within each experimental group. A Shapiro-Wilk test was employed to assess the normality of data distribution. Next, a statistical analysis of variance was performed with the ANOVA model, while significance (p-values) was determined by a Tukey's test at three levels of statistical significance ($p<0.05$; $p<0.01$; $p<0.001$).

We used K-means Cluster Analysis (KMCA) on representative Raman images to demonstrate its segmentation capability for intracellular structures. We used Manhattan distance and random centroid initialization to classify each Raman image into seven components: nucleus, perinuclear region, lipid droplets, cytochromes, two cytoplasmic components corresponding to thinner and thicker regions of the flattened cells, and the background. The same clustering parameters and seven-component classification scheme were applied consistently across the analyzed cell maps. We verified the stability of the classification by repeated analyses with random centroid initialization. The Raman spectra of KMCA classes were limited to 500-3050 cm^{-1}, baseline-corrected (10 iterations), smoothed using a 17-point Savitzky-Golay filter, and normalized by a unit vector. In some cases, vesicles originating from liquid-liquid phase separations with lower cytoplasmic density were identified by KMCA. These spectra were restricted to 500-3800 cm^{-1}, baseline-corrected (10 iterations), smoothed using a 9-point Savitzky-Golay filter, and normalized by unit vector relative to the O-H stretching band range of 3200-3800 cm^{-1}.

FTIR images were preprocessed in PCA-based noise reduction using 15 principal components and Savitzky-Golay smoothing (15 points). The mean spectrum of each cell was extracted using the region of interest (ROI) function, followed by application of the EMSC (Extended Multiplicative Signal Correction), baseline correction via the Automatic Whittaker Filter (asymmetry = 0.001 and $\lambda = 100$), calculation of the second derivative (polynomial order: 2, smoothing length: 13 points), and SNV normalization (Standard Normal Variate). Preprocessed spectra (100 per group) were subjected to unsupervised Principal Component Analysis (PCA) in the 3100-2800 and 1800-920 cm^{-1} regions using singular value decomposition with cross-validation and 7 principal components to achieve a 90% confidence level and avoid the noise contribution to data grouping. Scores and loadings plots were

generated to visualize cell grouping and spectral variation. Next, spectral data (100 spectra per group) were used to construct Partial Least Squares Discriminant Analysis (PLS-DA) and Support Vector Machine (SVM) models. In *Model 1*, the dataset comprised 100 spectra per experimental group (HBMEC, HA, and HBVP) from 3 biological and 2 technical replicates together, as described above. After random shuffling, the dataset was split into calibration (70%) and prediction (30%) sets using the Kennard–Stone algorithm with Euclidean distances. Cross-validation was performed using the Venetian blinds method with 10 data splits and one sample per blind. Support vector machine (SVM) classification was performed using a C-support vector classification (C-SVC) algorithm with a radial basis function (RBF) kernel. The optimal model parameters were determined to be cost = 100 and gamma = 0.31623. The final SVM model included 40 support vectors. *Model 2* was built using the monoculture data for calibration (n=100 cells per group) and the spectra from individual monocultures (n=100 per group) and HBMEC+HA+HBVP (100%, n=200 cells) for external prediction.

The O-PTIR ratio images for 1240/1740 cm^{-1} and 1740/2850 cm^{-1} were generated from single IR frequency images using Photothermal Corp software. The acquired hyperspectra were averaged before further analysis. The spectra were preprocessed using Savitzky-Golay smoothing (5 points, polynomial order 3), followed by constant normalization, baseline removal, and second-derivative calculation. Band integration of the pre-processed Raman and O-PTIR spectra was performed by calculating the area under the curve, and their intensities were normalized in the 0 – 1 range to construct heat maps.

SUPPORTING TABLES

Table S1. Primer sequences used for RT-qPCR analyses.

**RB1**	**F**	**CAGATGAAGCAGATGGAAGTAAACA**
	R	CGTGTTCGAGTAGAAGCTGGA
**RBL1**	F	AGCCGGAAGCAGAGGTAATTT
	R	GTGGCTCTTCAGAAGCCGTA
**CDK1**	F	TCAAAGCTGGCTCTTGGAAATTG
	R	AATGGGTATGGTAGATCCCGGC
**CCND1**	F	GATCAAGTGTGACCCGGACT
	R	CTTGGGGTCCATGTTCTGCT
**P21**	F	GGTCCAGCATGCTCCAGATTT
	R	ATGTAGAGCGGGCCTTTGAG
**CCNA2**	F	GGACCAGGAGAATATCAACCCG
	R	AGGGGTGCAACCCGTCTC
**CCNB1**	F	AACATCTGGATGTGCCCCTG
	R	TGGGCACACAATTATTCTCAAGT
**P53**	F	ATGGAGGAGCCGCAGTCAGAT
	R	CCATTGCTTGGGACGGCAAGG
**CCNE2**	F	TAGCTGGTCTGGCGAGGTTT
	R	GCCTGGATTATCTGGGCTTCT
**P27**	F	CCCTGAACGGAGCTGAAGTC
	R	TAACCGCGCAGCAGATAGTC
**TJP1**	F	CACCGGAGTCTGCCATTACA
	R	AACGTTGAATGCTTGCTGCT
**TJP3**	F	GTGGGGGCCGATTGACTG
	R	CTCCATGTCAGCCACCTGGAAT
**CLDN1**	F	CCCAGTCAATGCCAGGTACG
	R	ACACGTAGTCTTTCCCGCTG
**GAPDH**	F	GGAGCGAGATCCCTCCAAAAT
	R	GGCTGTTGTCATACTTCTCATGG

Table S2. Primary antibodies used for Western Blot analyses.

Target protein	Catalog No.	Type	Host species	Manufacturer	Location (city, country)
**Cyclin D1**	#2978	Monoclonal	Rabbit	Cell Signaling Technology	Danvers, MA, USA
**pRb (phospho-Retinoblastoma)**	#8516	Monoclonal	Rabbit	Cell Signaling Technology	Danvers, MA, USA
**p27 (CDKN1B)**	#3686	Monoclonal	Rabbit	Cell Signaling Technology	Danvers, MA, USA
**p21 (CDKN1A)**	#2947	Monoclonal	Rabbit	Cell Signaling Technology	Danvers, MA, USA
**Cyclin A2**	#4656	Monoclonal	Rabbit	Cell Signaling Technology	Danvers, MA, USA
**Cyclin B1**	#12231	Monoclonal	Rabbit	Cell Signaling Technology	Danvers, MA, USA
**p-cdc2 (phospho-CDK1 Tyr15)**	#4539	Monoclonal	Rabbit	Cell Signaling Technology	Danvers, MA, USA
**GFAP**	NBP2-33184F	Polyclonal	Mouse	Novus Biologicals	Centennial, CO, USA
**GAPDH (loading control)**	MAB374	Monoclonal	Mouse	Millipore / MilliporeSigma	Burlington, MA, USA

Table S3. Primary antibodies used for Flow Cytometry analyses.

Target protein / marker	Fluorochrome	Catalog No.	Type	Producer	Location (city, country)
**CD31**	BV785	303148	Monoclonal	BioLegend	San Diego, CA, USA
**CD140b (PDGFRβ)**	BV605	743035	Monoclonal	BD Biosciences	San Jose, CA, USA
**CD105 (Endoglin)**	PE	B92442	Monoclonal	Beckman Coulter	Brea, CA, USA
**CD45**	KrO (Krome Orange)	B36294	Monoclonal	Beckman Coulter	Brea, CA, USA
**P-gp (ABCB1)**	BV650	742850	Monoclonal	BD Biosciences	San Jose, CA, USA
