# Supplementary material for: Decoding Cell–Cell Interactions in Neurovascular Unit Co-Cultures Through Molecular Vibrational Spectroscopy and Biology
Source: Cells. 2026 Oct 15;15(20):1873. doi: 10.3390/cells15201873 (PMC13702744; doi:10.3390/cells15201873)
Supplement: Supplementary file 1 [file cells-15-01873-s001.zip › Interaction_Supplementary_Results.pdf]

Supplementary Information - Results

Decoding Cell–Cell Interactions in Neurovascular Unit Co-Cultures through Molecular Spectroscopy and Biochemical Analyses

Aleksandra Pragnąca[1,2], Agata Borkowska[3], Anna Antolak[1], Monika Leśniak[3], Karolina Augustyniak[1], Iga Czechowska[1], Kamilla Malek[1], Robert Zdanowski[3]

[1] Jagiellonian University in Krakow, Faculty of Chemistry, Department of Chemical Physics, Gronostajowa 2, 30-387, Krakow, Poland

[2] Jagiellonian University in Krakow, Doctoral School of Exact and Natural Sciences, prof. S. Lojasiewicza 11, 30-348 Krakow, Poland

[3] Military Institute of Medicine National Research Institute, Laboratory of Molecular Oncology and Innovative Therapies, Szaserow 128, 04-141 Warsaw, Poland

SUPPORTING TABLES 2

SUPPORTING FIGURES 4

REFERENCES 19

SUPPORTING TABLES

Table S4. Description of the PLS-DA models used to classify NVU cells and their associated performance metrics. *Model 1* was built using 70% of monoculture data (HBMEC, HA, HBVP) for calibration and 30% for prediction. *Model 2* was built using 100% of monoculture data for calibration and 100% of triple co-culture (HBMEC+HA+HBVP) for prediction.
Performance is reported using the following metrics: SeC – sensitivity for calibration; SpC – specificity for calibration; SeCV – sensitivity for cross-validation; SpCV – specificity for cross-validation; RMSEC – Root Mean Square Error of calibration; R^2C – coefficient of determination for calibration; RMSECV – Root Mean Square Error of cross-validation; R^2CV – coefficient of determination for cross-validation; RMSEP – Root Mean Square Error of prediction.

PLS-DA						
	Model 1			Model 2		
**Calibration group**	HBMEC = 70	HA = 70	HBVP = 70	HBMEC = 100	HA = 100	HBVP = 100
**Prediction group**	HBMEC = 30	HA = 30	HBVP = 30	HBMEC/HA/HBVP = 200		
**SeC**	1.000	1.000	1.000	1.000	1.000	1.000
**SpC**	0.993	1.000	0.993	1.000	1.000	0.995
**Class Error C**	0.004	0.000	0.004	0.000	0.000	0.003
**SeCV**	1.000	1.000	1.000	1.000	1.000	1.000
**SpCV**	0.993	1.000	0.986	1.000	1.000	0.995
**Class Error CV**	0.004	0.000	0.007	0.000	0.000	0.003
**RMSEC**	0.159	0.165	0.153	0.151	0.166	0.138
**R^2C**	0.886	0.878	0.895	0.897	0.876	0.914
**RMSECV**	0.164	0.172	0.159	0.154	0.170	0.143
**R^2CV**	0.878	0.867	0.886	0.897	0.869	0.908
**RMSEP**	0.100	0.940	0.976	0.090	0.922	0.965

Table S5. Description of the SVM model used to classify NVU cells and their associated performance metrics. *Model 1* was built using 70% of monoculture data (HBMEC, HA, HBVP) for calibration and 30% for prediction.
Performance is reported using the following metrics: SeC – sensitivity for calibration; SpC – specificity for calibration; SeCV – sensitivity for cross-validation; SpCV – specificity for cross-validation.

SVM			
	Model 1		
**Calibration group**	HBMEC = 70	HA = 70	HBVP = 70
**Prediction group**	HBMEC = 30	HA = 30	HBVP = 30
**SeC**	0.971	1.000	0.986
**SpC**	0.993	1.000	0.986
**Class Error C**	0.018	0.000	0.014
**SeCV**	0.957	1.000	0.943
**SpCV**	0.971	1.000	0.979
**Class Error CV**	0.036	0.000	0.039

SUPPORTING FIGURES

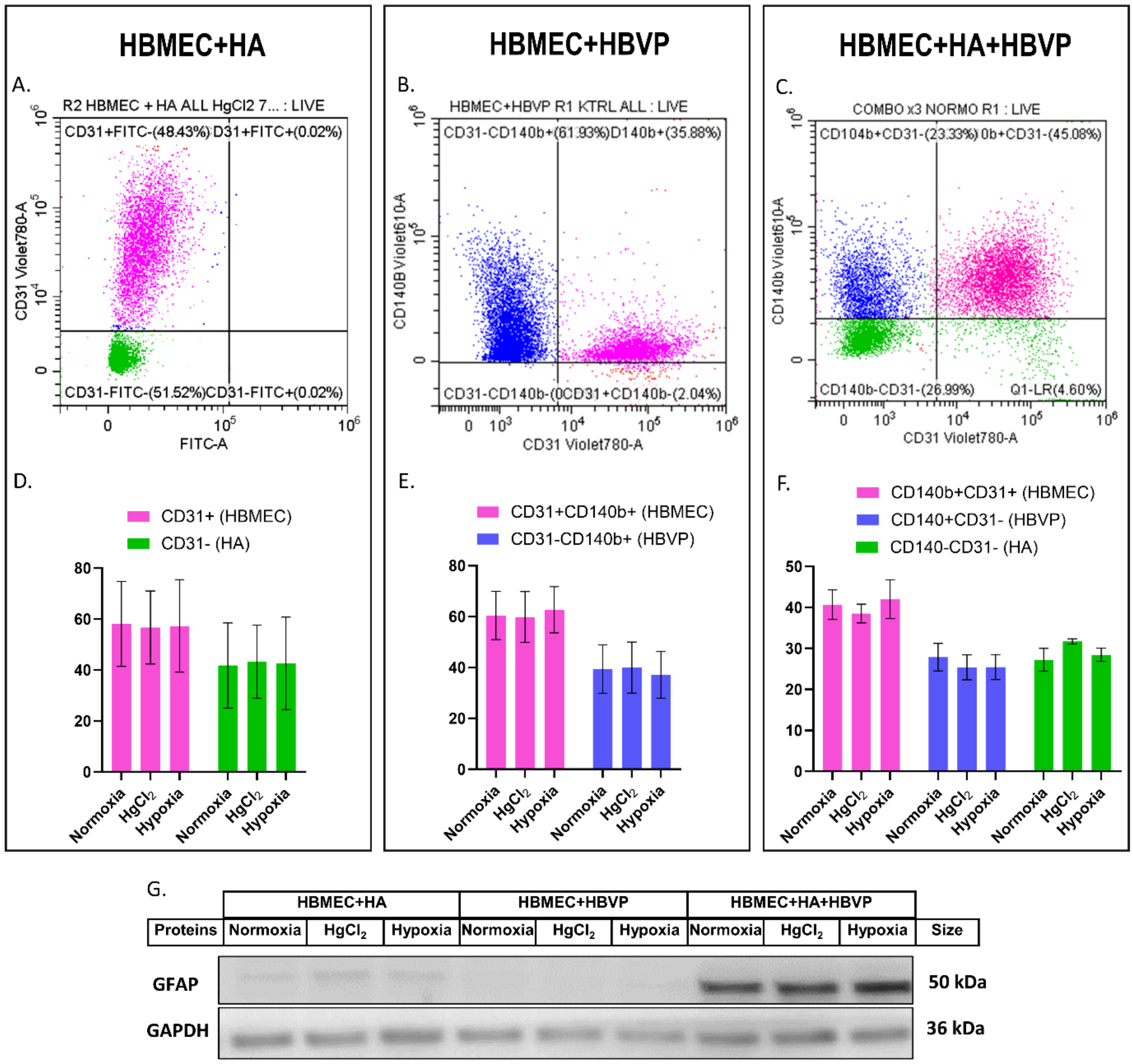

Figure S1. Molecular characterization of NVU systems. (**1**). Surface expression of cellular markers for (**A**) HBMEC+HA - CD31 (magenta) and FITC (green), (**B**) HBMEC+HBVP – CD140b (blue) and CD31 (magenta), and (**C**) HBMEC+HA+HBVP - CD140b CD140b (blue) and CD31 (magenta). Data shown are representative of n≥3 experiments. Distribution of NVU cell types in co-cultures: (D) HBMEC+HA, (E) HBMEC+HBVP and (F) HBMEC+HA+HBVP. Daya presented as mean±SEM for n≥3.

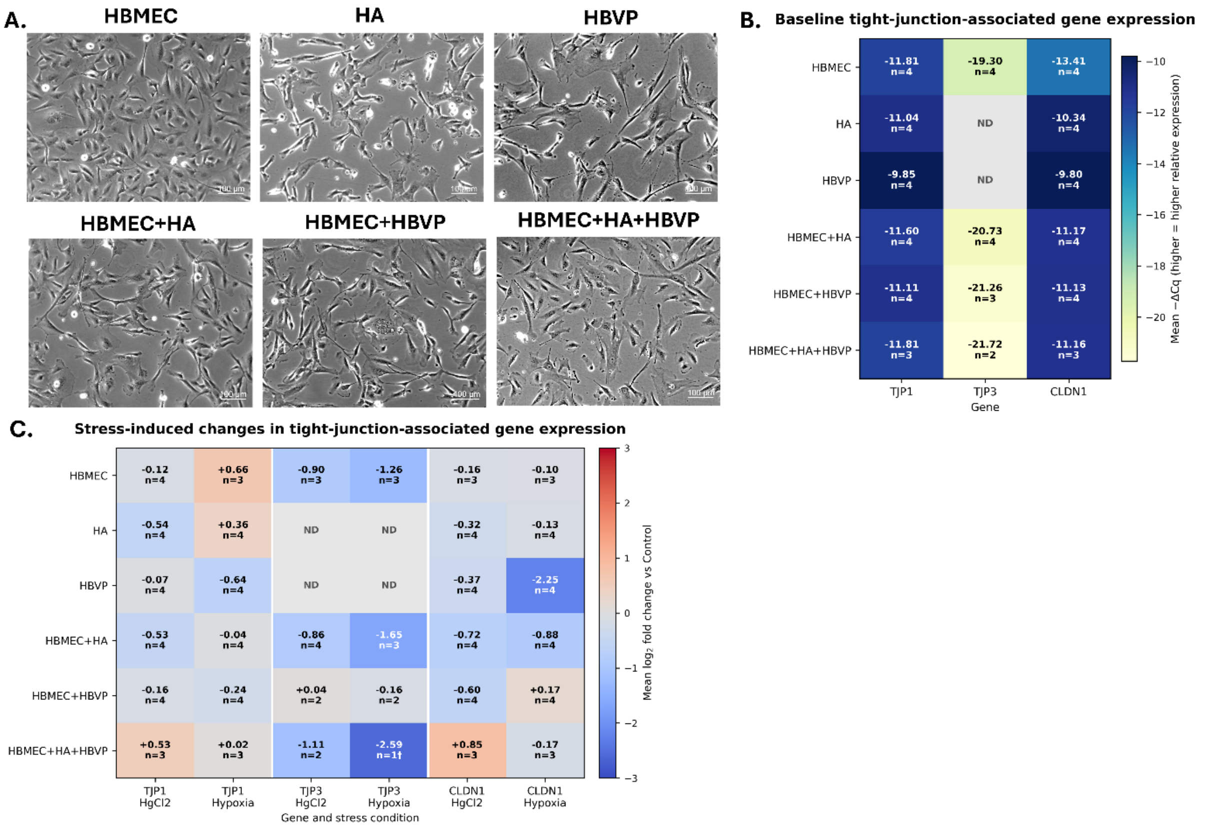

Figure S2. Morphological and molecular characterization of the investigated NVU systems. (A) Representative transmitted-light images of HBMEC, HA, HBVP, HBMEC+HA, HBMEC+HBVP, and HBMEC+HA+HBVP cultures under control conditions, illustrating the sub-confluent organization used for spatially resolved spectroscopic analysis; (B) Baseline expression of tight-junction-associated genes TJP1, TJP3, and CLDN1 under control conditions, expressed as mean −ΔCq relative to GAPDH/PPIA. Higher −ΔCq values indicate higher relative transcript abundance; (C) Stress-induced changes in TJP1, TJP3, and CLDN1 expression following $HgCl_2$ exposure or hypoxia, expressed as mean log_2 fold change relative to matched controls. n indicates paired biological replicates; ND, not quantifiable.

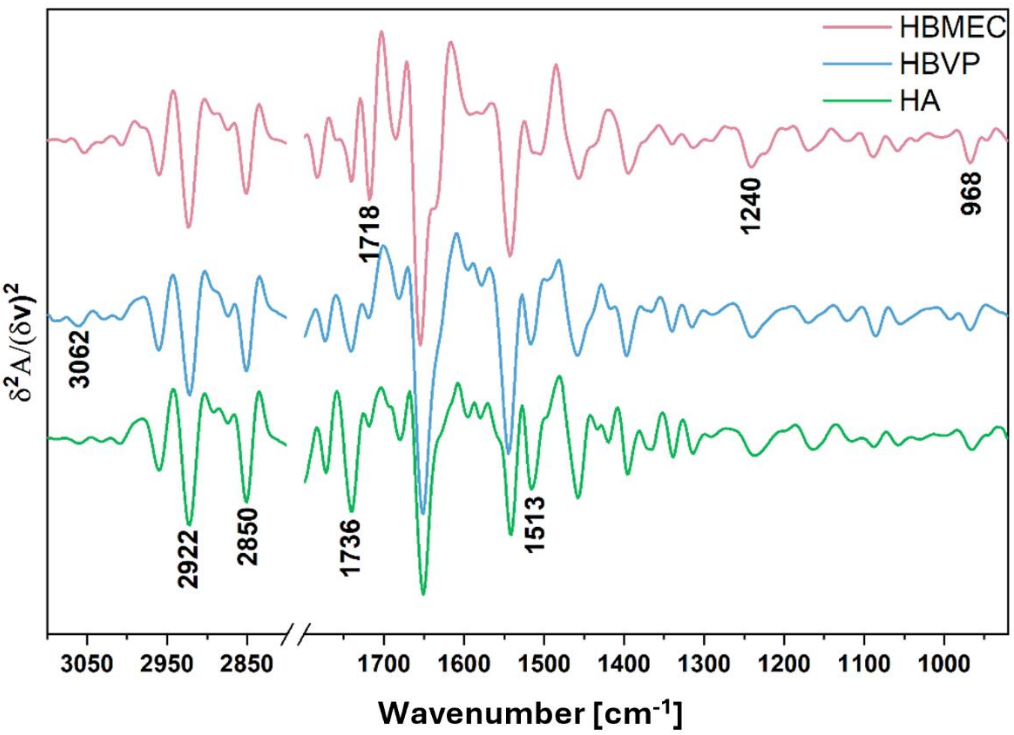

Figure S3. The average second derivative FTIR spectra (N = 100) of HBMEC, HBVP, and HA cell lines cultured individually. The spectra are displayed in the bioregion of 920-1800 and 2800-3100 cm^{-1}, with bands characteristic of each cell line and identified in the PCA loadings plots in **Fig. 2.1**.

1 A

	HBMEC	HA	HBVP
Predicted as HBMEC	68	0	0
Predicted as HA	0	70	0
Predicted as HBVP	0	0	66
Predicted as Unasigned	2	0	4

B

	HBMEC	HA	HBVP
Predicted as HBMEC	67	0	0
Predicted as HA	0	70	4
Predicted as HBVP	3	0	66
Predicted as Unasigned	0	0	0

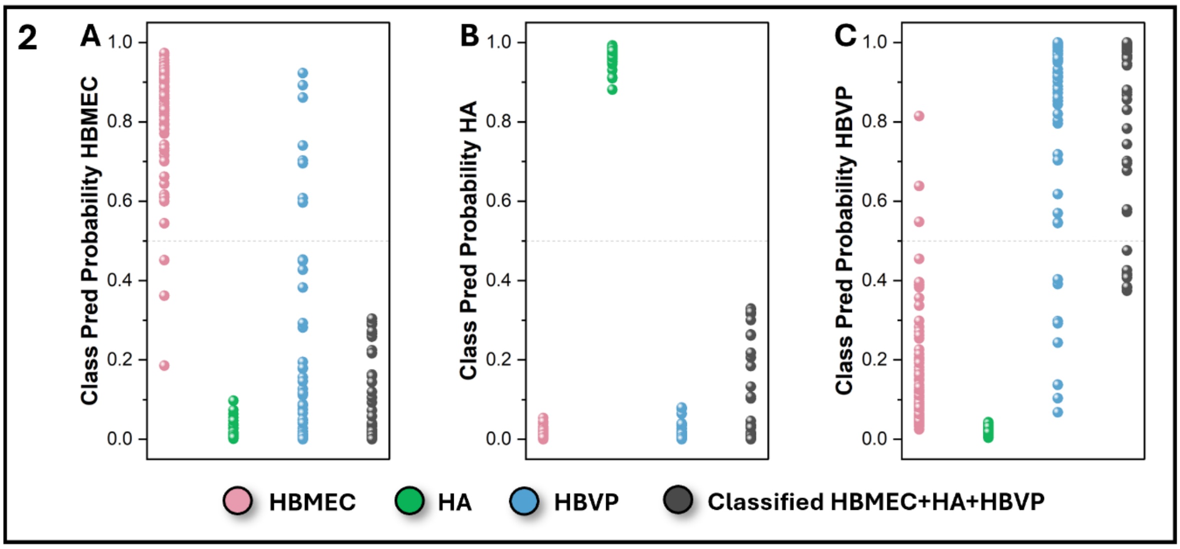

Figure S4. Classification results. Panel 1. Confusion matrices for Model 1 calculated using (A) PLS-DA and (B) SVM. Panel 2. Predicted SVM classification probabilities of HBMEC+HA+HBVP (Model 2) for (A) HBMEC, (B) HA, and (C) HBVP. The dashed horizontal line represents a probability of 0.5.

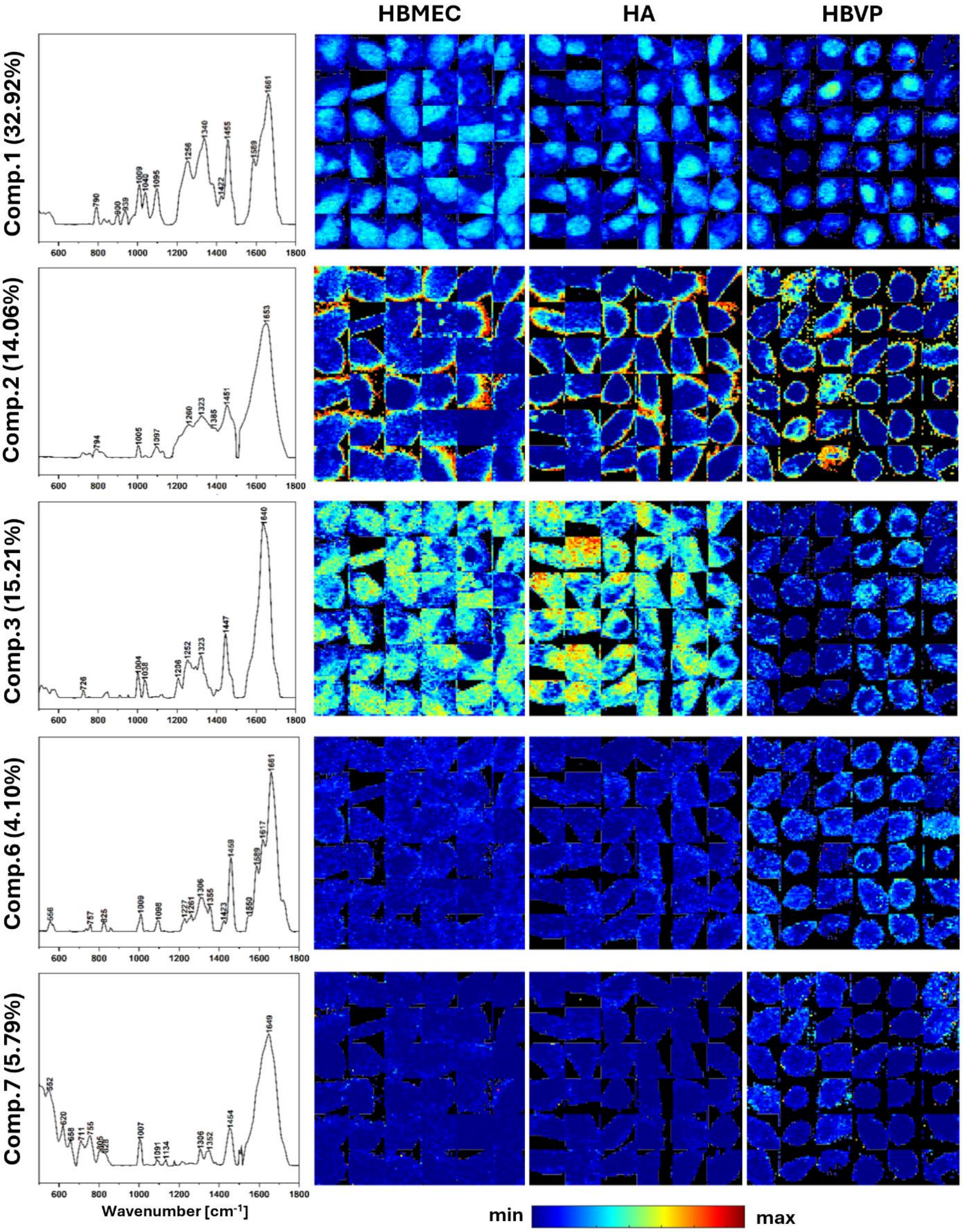

Figure S5. MCR analysis calculated from Raman images of the monocultures of HBMEC, HA, and HBVP in **normoxic conditions**, with remaining loading plots of distinguished components (*left*) and their distribution (*right*). MCR components were calculated from representative 36 images in the fingerprint region (500-1800 cm^{-1}).

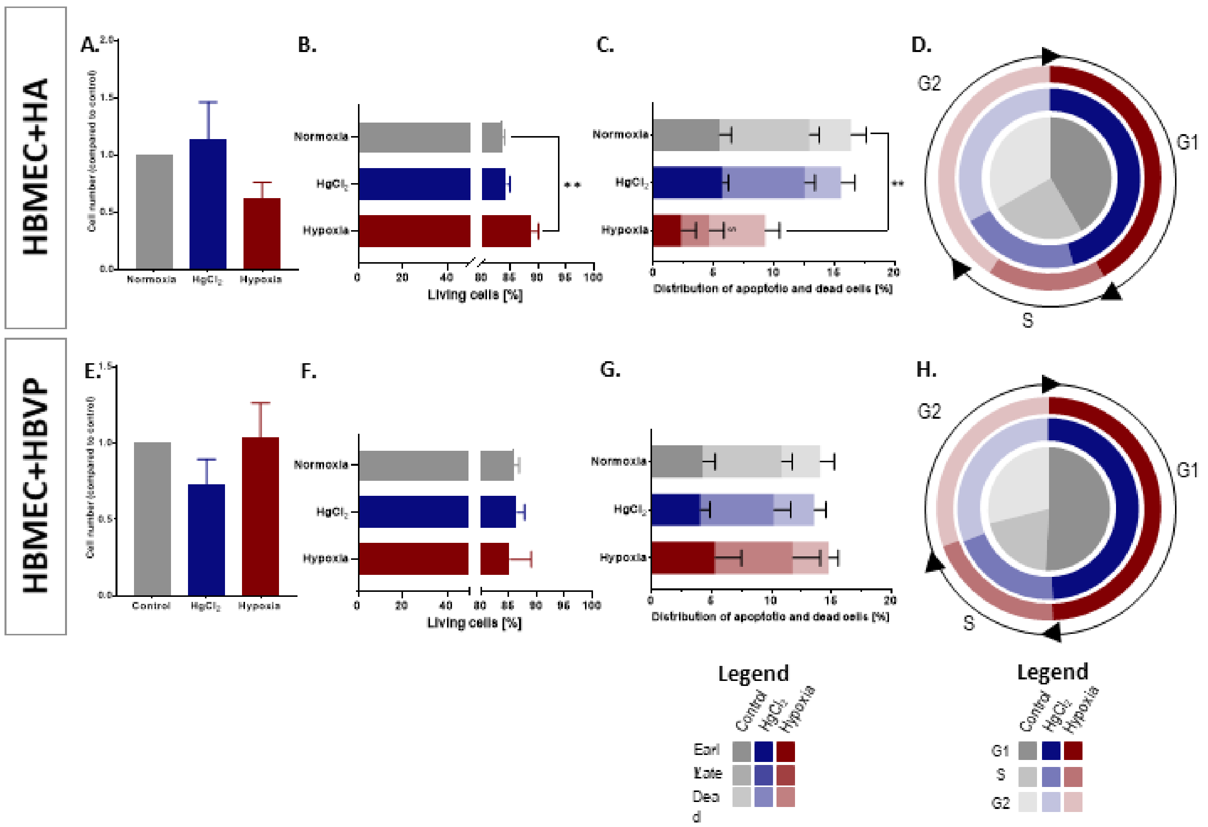

Figure S6. An effect of stress on cell number, viability, apoptosis, and cell cycle distribution in double co-cultures. Fold change in total cell number relative to normoxia for HBMEC +HA (**A**) and HBMEC+HBVP (**E**). Viability of cells (percentage of living cells) HBMEC +HA (**B**) and HBMEC+HBVP (**F**). Distribution of early apoptotic (dark grey/red/blue), late apoptotic (medium grey/red/blue), and dead cells (light grey/red/blue) among non-viable populations of HBMEC +HA (**C**) and HBMEC+HBVP (**G**). Cell cycle phase distribution based on flow cytometry analysis of DNA content in HBMEC +HA (D) and HBMEC+HBVP (**H**). Proportions of cells in G_1 (dark gray/red/blue), S (middle grey/red/blue), and G_2 (light grey/red/blue) phases are depicted as circular plots. Statistical significance is indicated as relative to the normoxic control: $/*$p<0.05$. Each result is presented as mean ± SEM, except for cell cycle distribution, where results are presented as mean. Each experiment was performed n≥3.

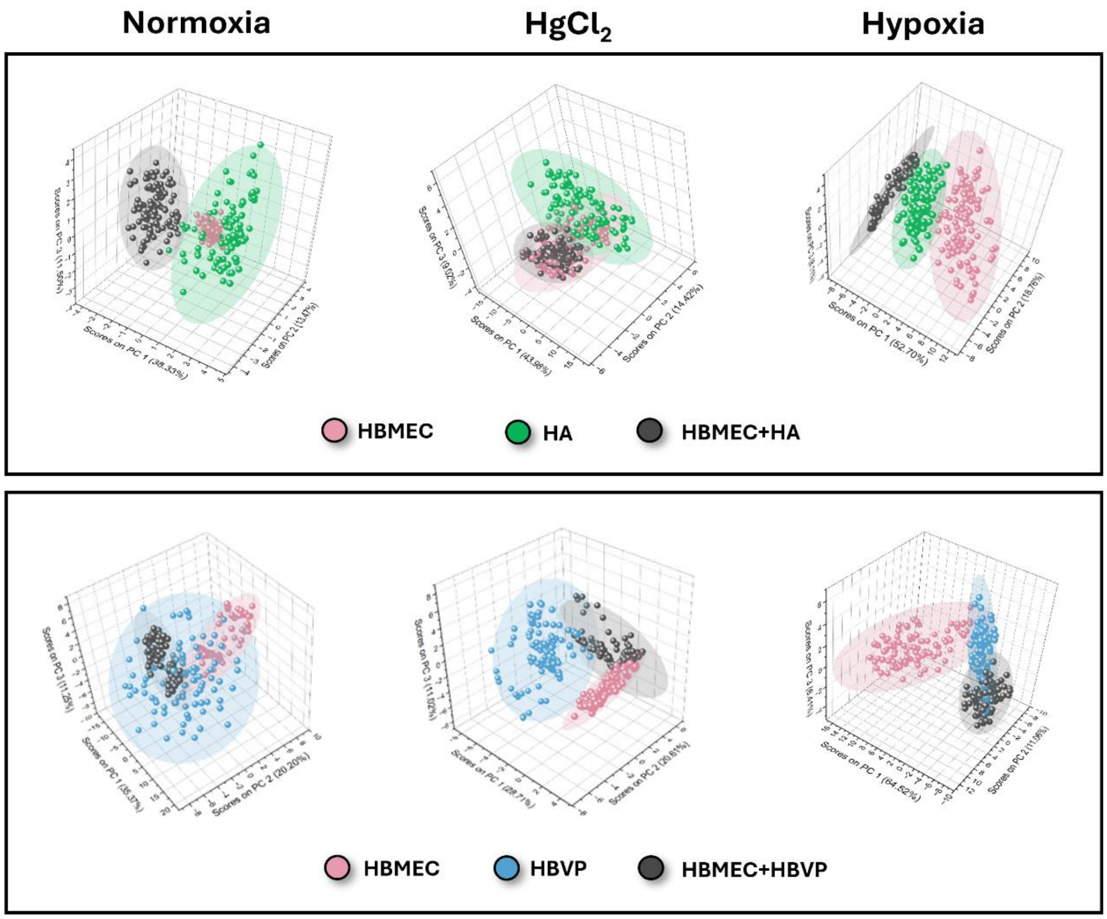

Figure S7. IR phenotyping of the stress response in the NVU monocultures and double co-cultures. The scores plots were generated from whole-cell FTIR spectra of 100 cells/group in the bioregion (920-1800 and 2800-3100 cm^{-1}). Each point corresponds to one cell.

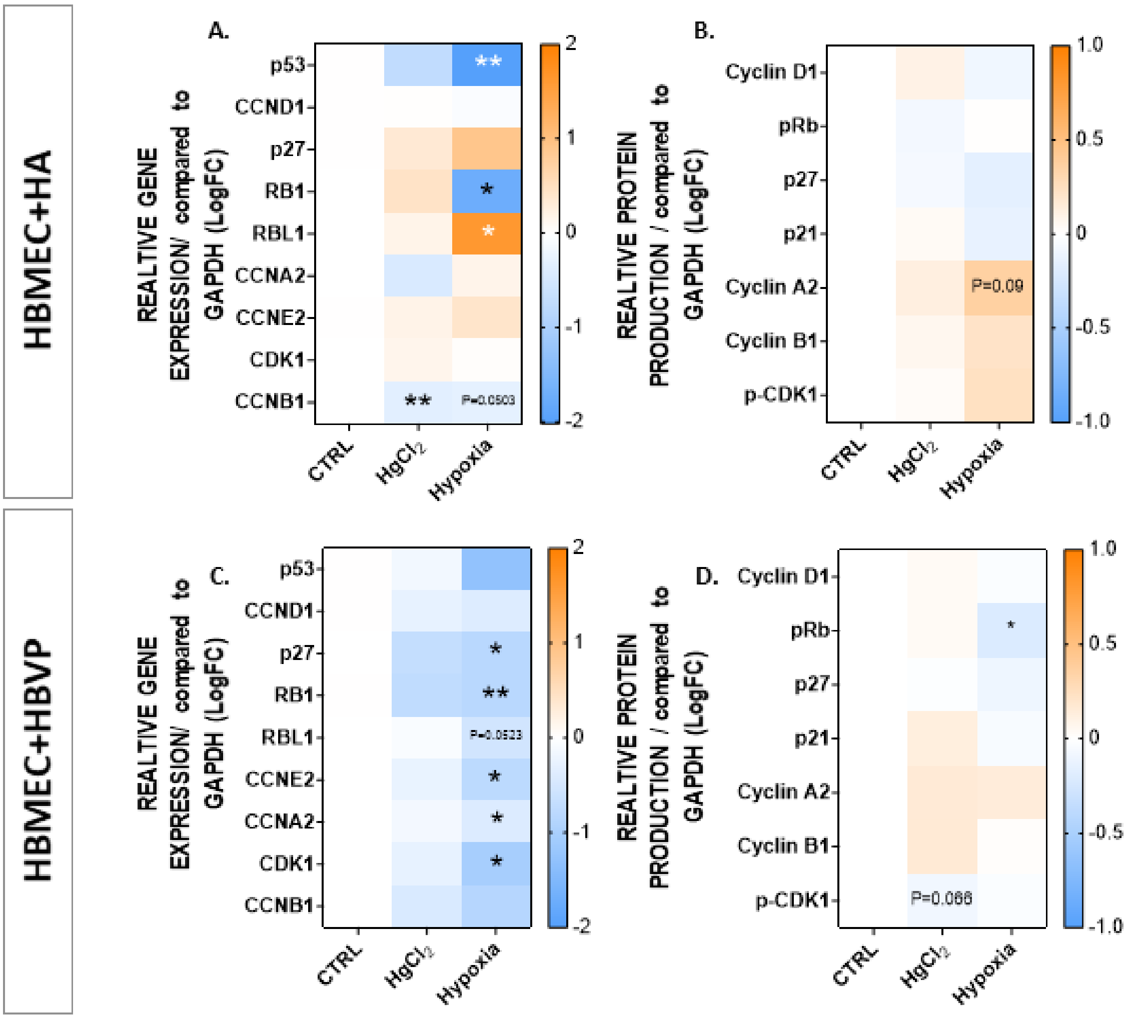

Figure S8. Stress exposure triggers distinct cell cycle responses in different NVU systems. Heatmaps of (**A, C**) mRNA fold-changes for key cell cycle regulators (p53, CCND1, p27, RB1, RBL1, CCNE2, CCNA2, CDK1, CCNB1) and (**B, D**) relative protein production in comparison to GAPDH presented as fold changes (Cyclin D1, pRb, p27, p21, Cyclin A2, Cyclin B1, p-cdc2) in **(A-B)** HBMEC+HA **(C-D)** HBMEC+HBVP. Each box represents the mean value from n≥3 experiments. Asterisks indicate statistical significance in comparison to normoxic control (*p<0.05; **p<0.01).

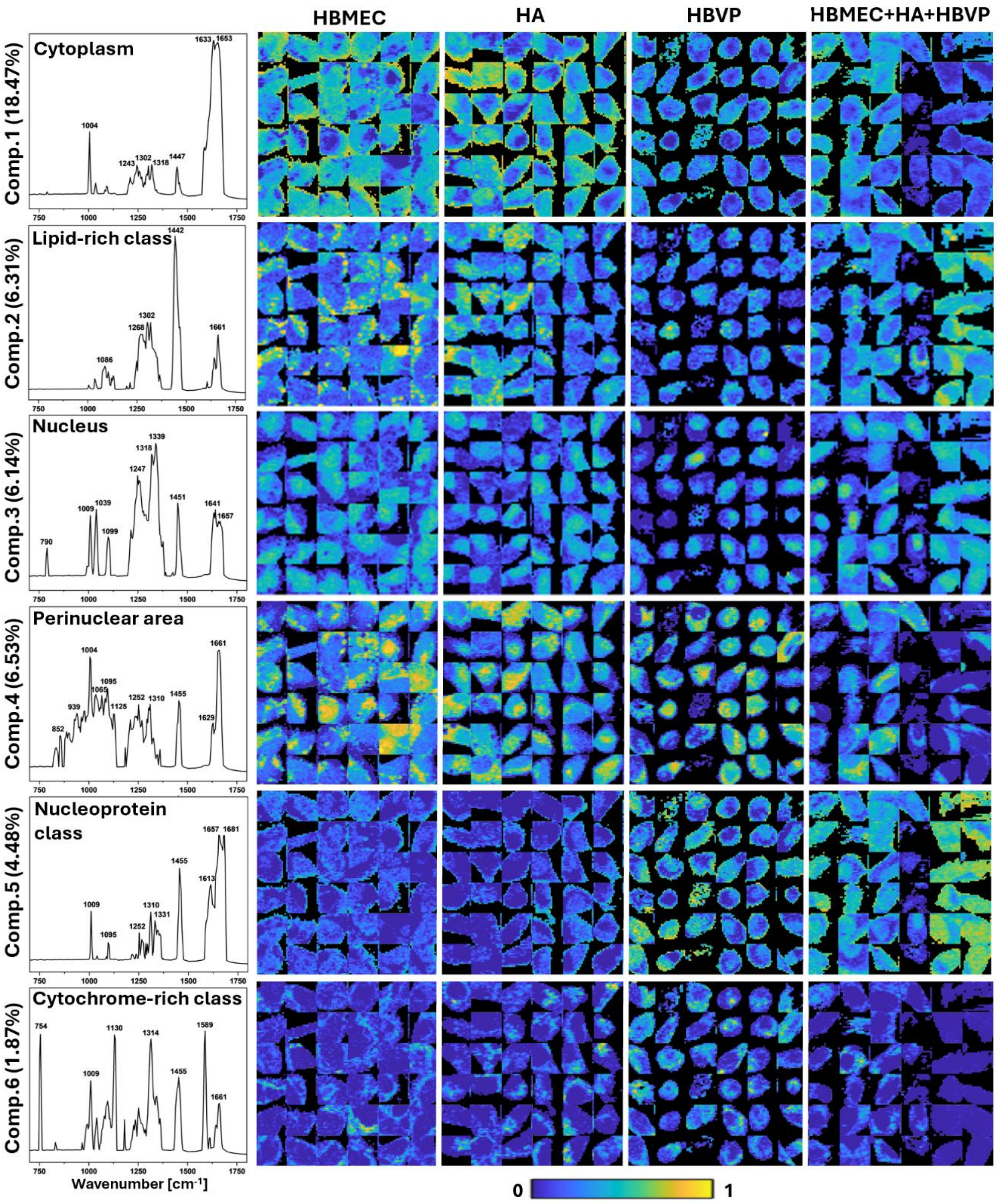

Figure S9. MCR analysis calculated from Raman images of the monocultures (HBMEC, HA, and HBVP) and their triple co-culture (HBMEC+HA+HBVP) in **normoxic conditions**, with loading plots of distinguished components (*left*) and their distribution (*right*). MCR components were calculated from representative 36 images in the fingerprint region (500-1800 cm^{-1}). All component values were normalized to the (0-1) range. Raman bands labelled in the plots are specific to each cellular compartment [37].

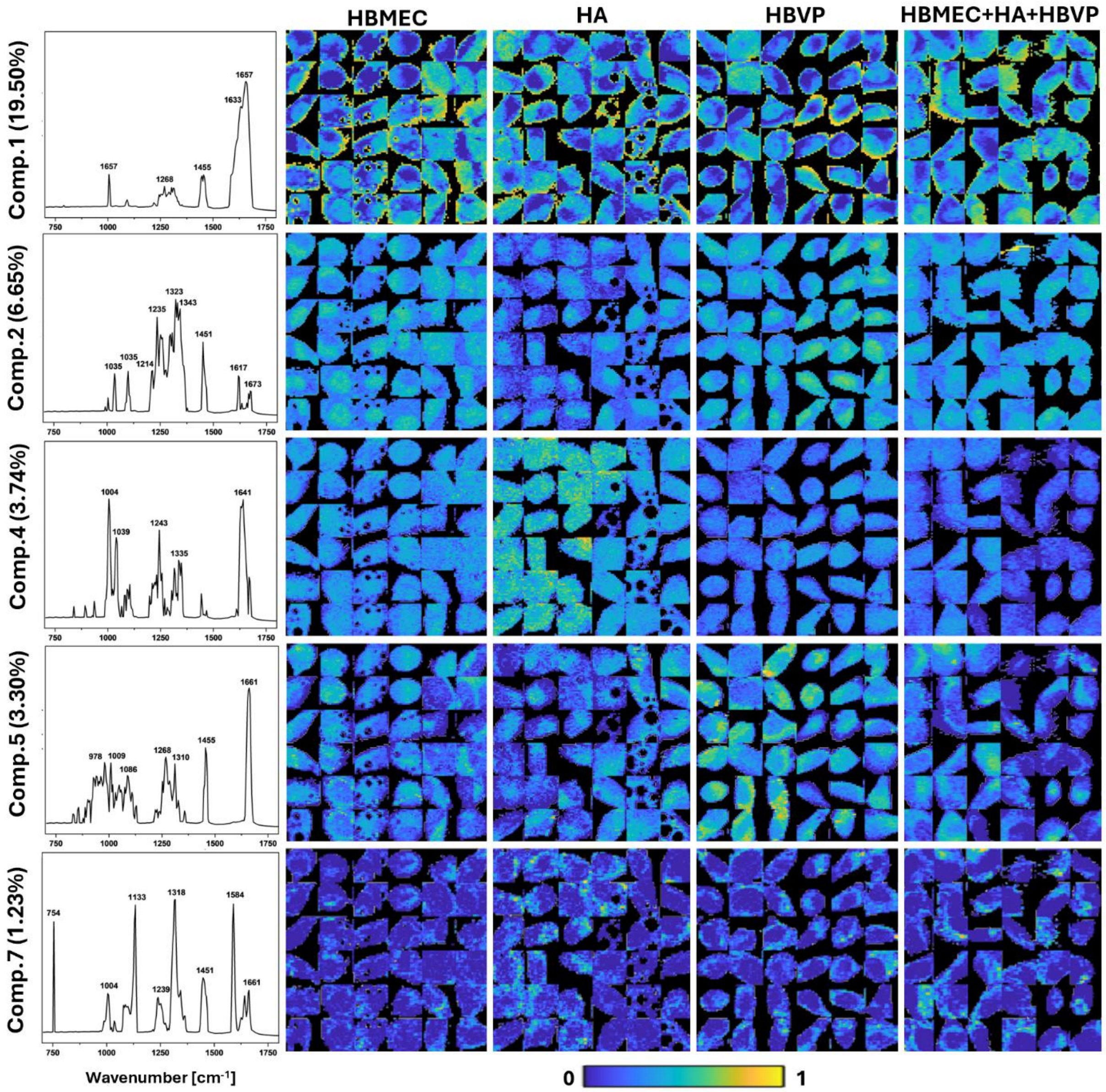

Figure S10. MCR analysis calculated from Raman images of the monocultures (HBMEC, HA, and HBVP) and their triple co-culture (HBMEC+HA+HBVP) in **$HgCl_2$ conditions**, with loading plots of remaining distinguished components (*left*) and their distribution (*right*). MCR components were calculated from representative 36 images in the fingerprint region (500-1800 cm^{-1}). All component values were normalized to the (0-1) range.

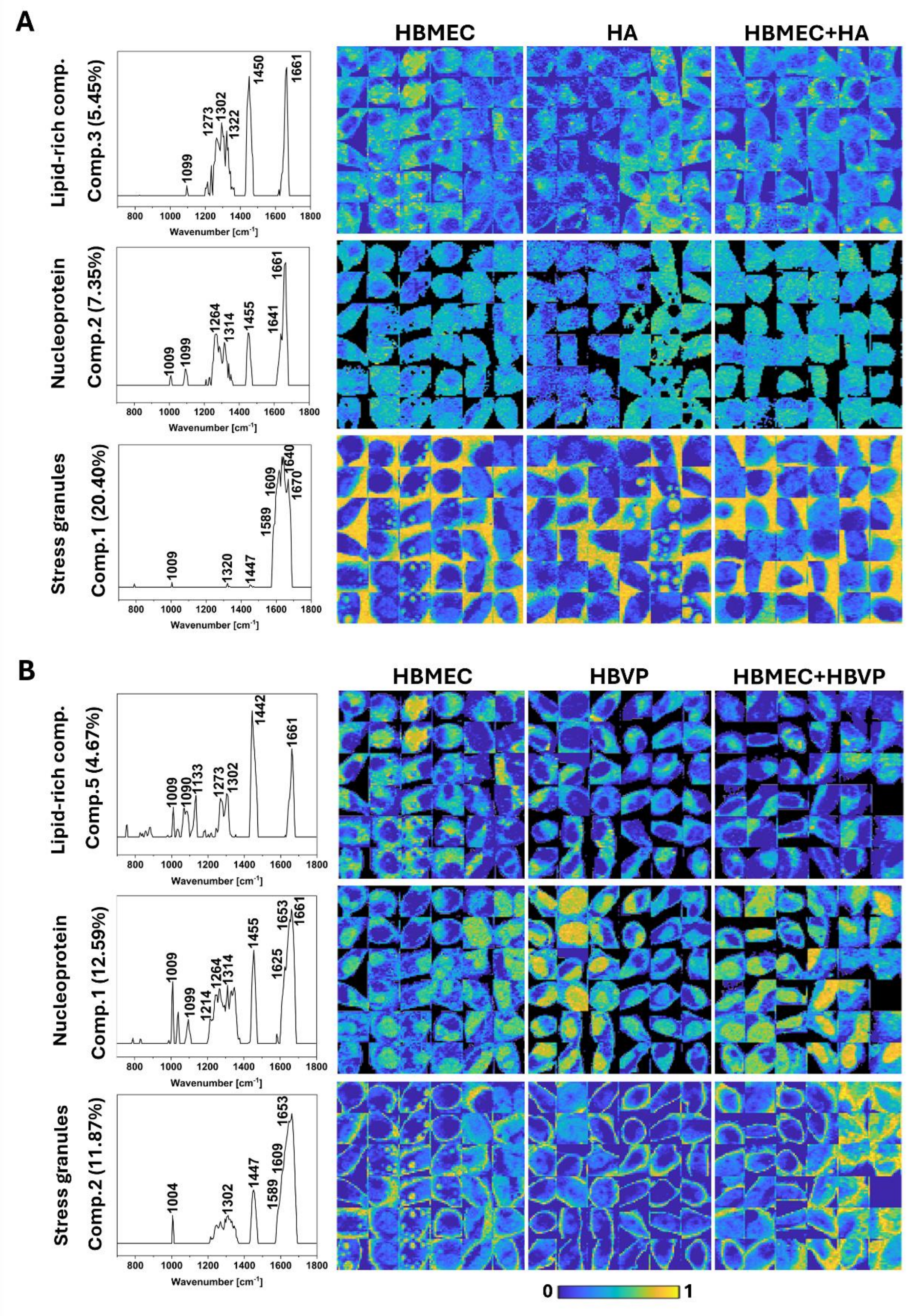

Figure S11. Selected MCR analysis calculated from Raman images of the di-cultures of (**A**) HBMEC+HA and (**B**) HBMEC+HBVP in **$HgCl_2$ conditions**, with loading plots of distinguished components (*left*) and their distribution (*right*). MCR components were calculated from representative 36 images in the fingerprint region (500-1800 cm^{-1}). All component values were normalized to the (0-1) range.

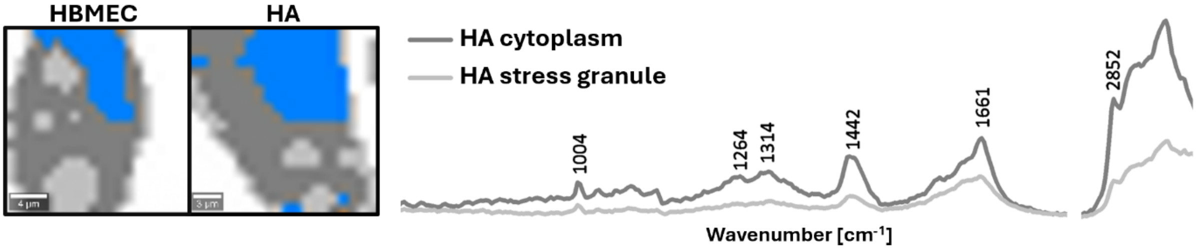

Figure S12. KMCA maps of the HBMEC and HA cells (*left*), identifying **stress granules** (**SGs**; light grey) localized in the cytoplasm (dark grey), and nucleus (blue). Exemplary Raman spectra of SGs and adjacent cytoplasm in HA (*right*) indicate the protein profile of SGs.

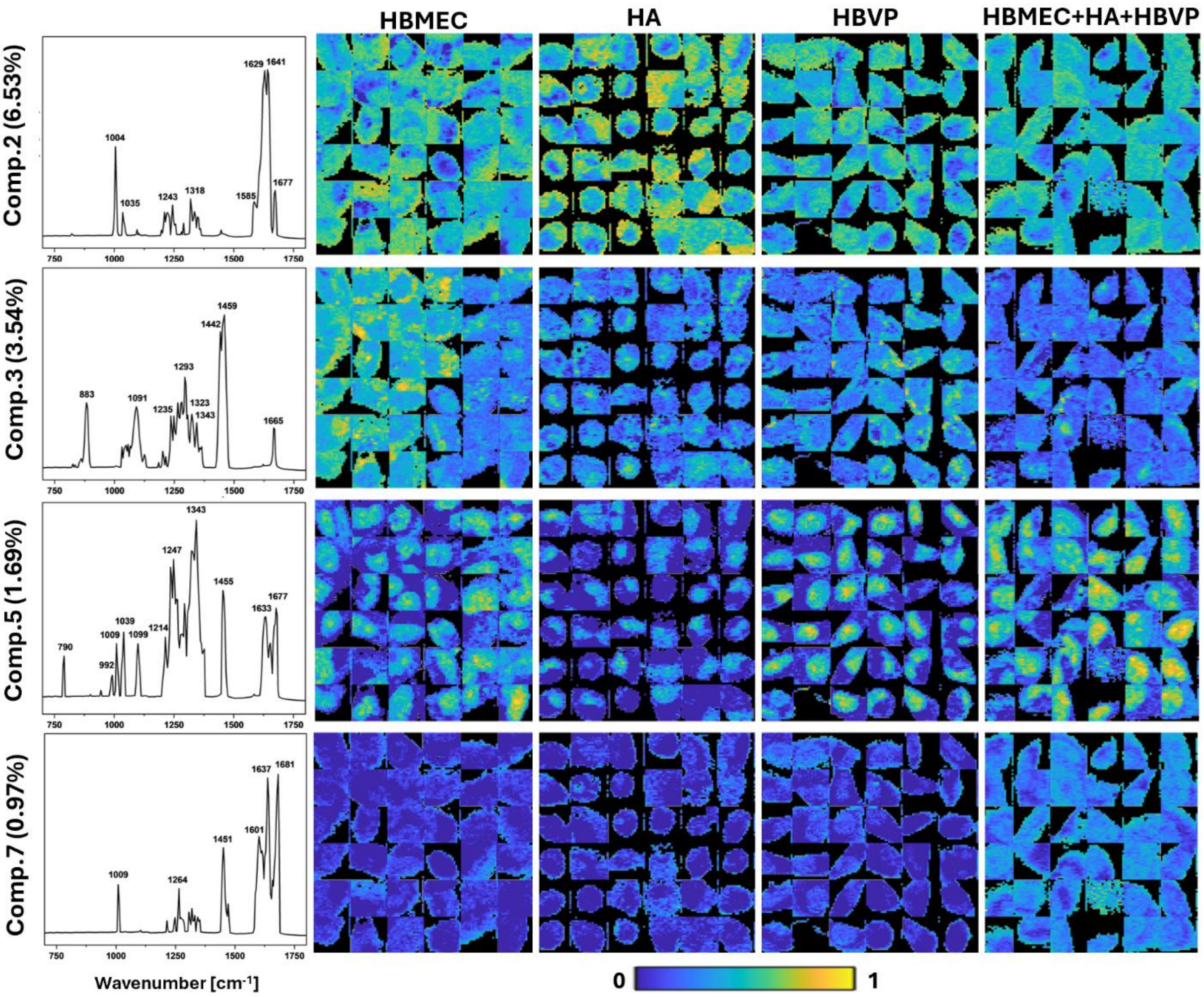

Figure S13. MCR analysis calculated from Raman images of the monocultures (HBMEC, HA, and HBVP) and their triple co-culture (HBMEC+HA+HBVP) in **hypoxic conditions**, with loading plots of remaining distinguished components (*left*) and their distribution (*right*). MCR components were calculated from representative 36 images in the fingerprint region (500-1800 cm^{-1}). All component values were normalized to the (0-1) range.

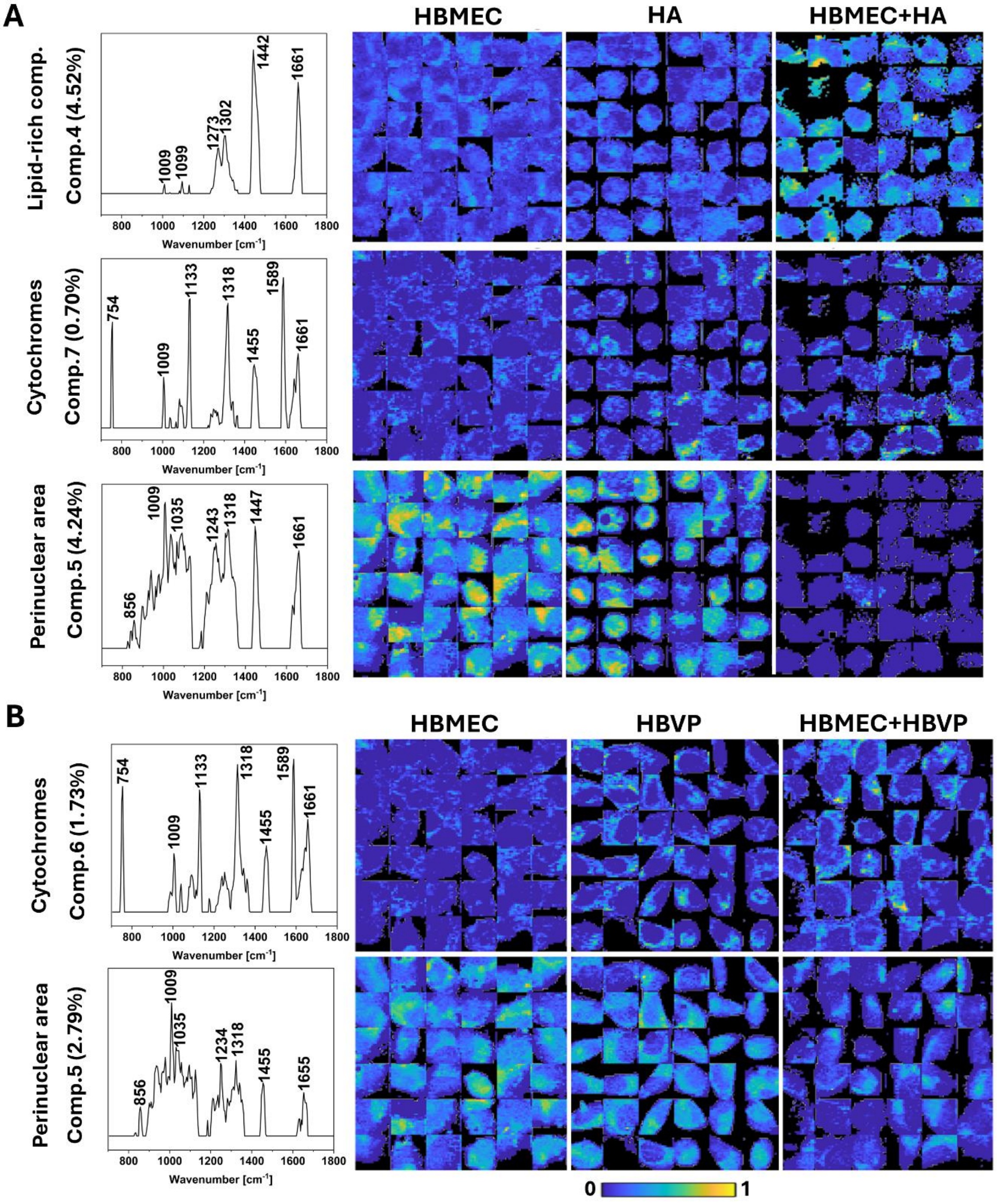

Figure S14. Selected MCR analysis calculated from Raman images of the di-cultures of (**A**) HBMEC+HA and (**B**) HBMEC+HBVP in **hypoxic conditions**, with loading plots of distinguished components (*left*) and their distribution (*right*). MCR components were calculated from representative 36 images in the fingerprint region (500-1800 cm^{-1}). All component values were normalized to the (0-1) range.

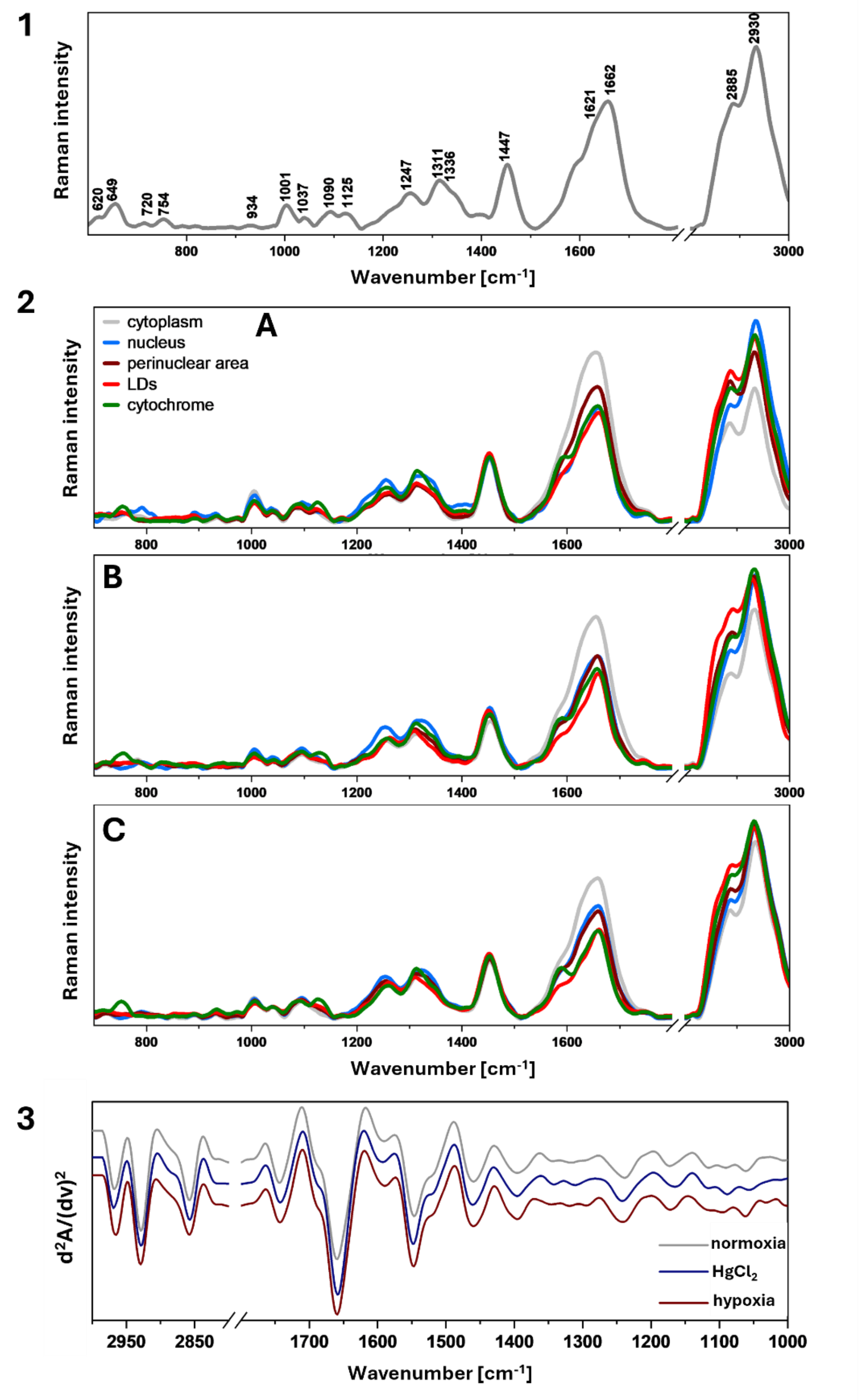

Figure S15. (**1**) Average Raman spectra extracted using K-means cluster analysis (KMCA) from filopodia of cells engaged in physical contact under normoxic conditions show strong similarity to that of purified actin [39]. (**2**) Exemplary KMCA mean Raman spectra of classes determined in cells creating physical contact in (**A**) normoxia and affected by (**B**) $HgCl_2$ and (**C**) hypoxia. The colors of the spectra correspond to the colors of the classes in false-color KMCA maps in **Figure 6**. (**3**) Second derivative OPTIR spectra from TAG-rich pixels in triple co-culture of BBB cells.

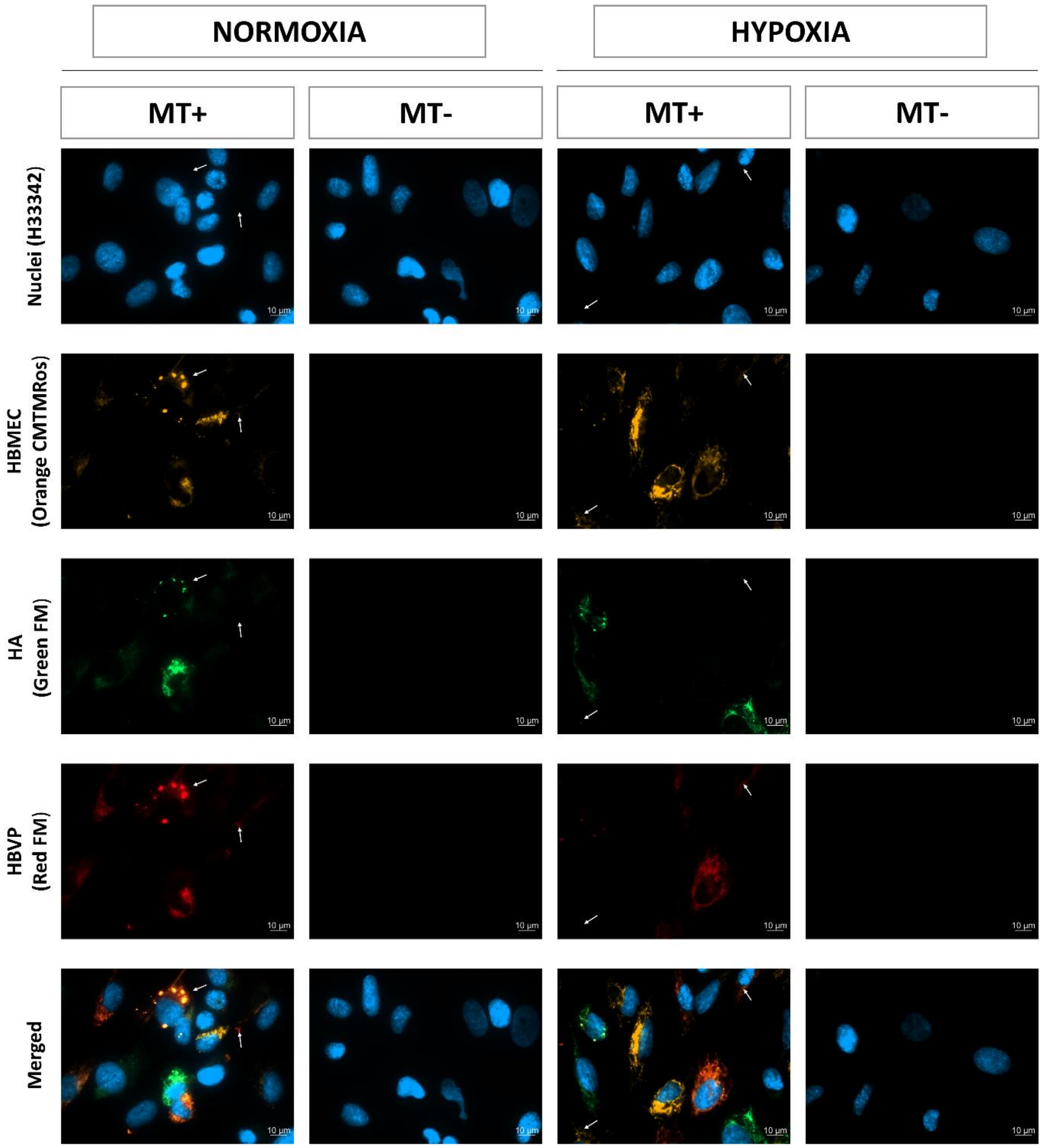

Figure S16. Fluorescence microscopy images of HBMEC+HA+HBVP co-cultures labelled with MitoTracker dyes under normoxic and hypoxic conditions. Cells were labelled with distinct MitoTracker dyes prior to seeding: HBMEC – orange, HA – green and HBVP – red. Nuclei were stained with H33342. Cells were cultured under normoxic or hypoxic conditions. Representative fluorescence images of labeled (MT+) and unlabeled cells (MT-) were acquired at 63× magnification objective. Scale bar = 10 µm.

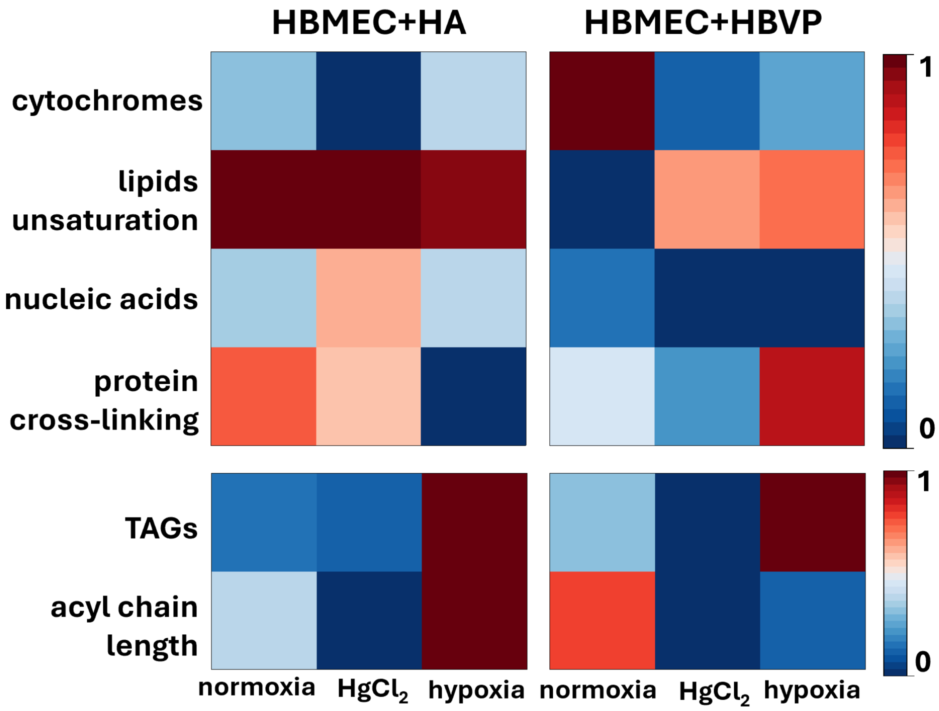

Figure S17. Heat maps of stress markers in di-cultures calculated from band intensities of cytochromes (**RS**: ratio of 750/1440 cm^{-1}), lipid unsaturation (**RS** (LDs): ratio of 1660/1440 cm^{-1}), protein crosslinking (**RS** (cytoplasm): ratio of 1040/1005 cm^{-1}), nucleic acids (**RS** (cytoplasm): ratio of 790/1440 cm^{-1}), TAGs and acyl chain length in fatty acids (**O-PTIR** (TAG-rich pixels): ratios of 1740/2850 and 2850/2960 cm^{-1}, respectively). The values were normalized to 0-1.

REFERENCES

37. Pragnąca, A.; Antolak, A.; Krysiak, Z.J.; Leśniak, M.; Borkowska, A.; Zdanowski, R.; Malek, K. Marker-Independent Vibrational Spectroscopy Imaging Recognizes the Hypoxia Effect in the Human Brain Endothelium. *Sci. Rep.* **2025**, *15*, 1–13, doi:10.1038/s41598-025-11000-2.

39. de Jong, B.W.D.; Bakker Schut, T.C.; Wolffenbuttel, K.P.; Nijman, J.M.; Kok, D.J.; Puppels, G.J. Identification of Bladder Wall Layers by Raman Spectroscopy. *J. Urol.* **2002**, *168*, 1771–1778, doi:10.1097/01.ju.0000030059.28948.c6.
